# Optimized passive thermoelectric temperature regulation for dust-resilient sensor nodes in Extreme Desert Climates

**DOI:** 10.1371/journal.pone.0352185

**Published:** 2026-06-26

**Authors:** Md. Mahib Al Mamun, Rafin Ahmed, Oishik Arham Audhip, Hassan Jubair

**Affiliations:** 1 HR Institute, Khulna, Bangladesh; 2 Khulna University of Engineering and Technology, Khulna, Bangladesh; Qingdao University, CHINA

## Abstract

Autonomous sensor nodes deployed in harsh, remote desert environments face significant reliability challenges due to extreme diurnal temperature fluctuations. This paper presents an Optimized Passive Thermoelectric Temperature Regulation System designed to protect sensitive electronics and batteries from these conditions. The innovative system leverages a solid-state Peltier module for active bi-directional temperature control (cooling and heating), crucially supported by novel, highly efficient fanless passive heat transfer components. The system integrates thermoelectric regulation with optimized passive heat dissipation and solar-powered energy management, enabling continuous 24/7 operation. Experimental validation under simulated desert environments demonstrates a coefficient of performance (COP) of up to 0.83, cooling from approximately 40°C to 20°C within 4.6 minutes, and approximately 10% reduction in power consumption compared to forced-convection systems. Its holistically designed, sealed, and dust-proof enclosure, devoid of mechanical moving parts in the thermal management system (fans, compressors), drastically enhances reliability and minimizes maintenance requirements compared to traditional methods like forced air cooling or miniature vapor-compression systems. This approach uniquely addresses the compounded challenges of dust, extreme temperatures, and limited power. It offers a superior solution for ensuring the uninterrupted accuracy, extended lifespan, and demonstrated improved performance over conventional mechanisms of vital sensor nodes in arid climates.

## 1. Introduction

In the evolving landscape of autonomous sensor node deployment, particularly within harsh remote desert environments, thermal management remains a critical and unresolved engineering challenge. These environments impose extreme diurnal temperature swings, often exceeding 50°C during the day and plummeting below freezing at night. This places significant thermal stress on sensitive electronics and energy storage systems. Such fluctuations compromise the operational integrity, reliability, and lifespan of sensor nodes expected to function autonomously without maintenance for extended periods [1,2].

Elevated temperatures are a dominant factor in system degradation. Research attributes over 50% of electronic failures directly to thermal stress [3], a challenge validated by recent condition monitoring studies [4,5]. Statistically, a mere 10°C increase in internal temperature can halve the reliability of electronic components and reduce capacitor lifespan by up to 50% [6]. Fans, often the default choice for active cooling, also experience sharply reduced longevity in high-temperature environments. For every 10°C rise, bearing wear accelerates dramatically, compromising system uptime and increasing failure rates [7,8]. Concurrently, batteries subjected to ambient temperatures above 35°C suffer irreversible reductions in capacity and charge retention, undermining the power autonomy essential for remote operations [9,10].

Conventional cooling strategies are fundamentally inadequate for such conditions. Forced-air cooling systems are highly vulnerable to fine desert dust and sand. These particulates quickly clog filters, abrade fan components, and severely reduce convective heat transfer efficiency [11]. Furthermore, the sweltering desert air minimizes the temperature differential needed for efficient heat dissipation via convection, rendering these systems thermally inefficient [12]. Conversely, purely passive cooling mechanisms depending solely on natural convection and radiation are inherently limited by ambient conditions. They cannot actively cool below external temperatures or provide necessary heating during freezing nights [13]. Miniaturized vapor-compression systems offer active thermal control but introduce mechanical complexity and maintenance demands unsuitable for remote deployment. Components like compressors and seals are prone to vibration-induced failure and refrigerant leakage, risks exacerbated by harsh operating conditions [14].

To address these critical limitations, this paper proposes the Optimized Passive Thermoelectric Temperature Regulation System (OPTERS) tailored for autonomous deployment in desert environments. This system exploits the solid-state characteristics of Peltier modules [15] to enable bidirectional thermal regulation (both active cooling and heating) without reliance on mechanical parts vulnerable to dust, wear, or vibration. By integrating optimized passive heat sinks and fanless thermal interfaces, the design ensures efficient heat exchange while eliminating the failure modes associated with conventional active systems.

Critically, the OPTERS solution balances performance, robustness, and cost-efficiency. Utilizing inexpensive, widely available components, including Peltier modules, ESP32 microcontrollers, and repurposed highly conductive materials, the system achieves reliable operation with minimal maintenance (illustrated in Fig 2). Its low power consumption and the absence of mechanically intensive parts significantly reduce both the initial deployment cost and long-term operational expenditures. As a result, this approach offers a resilient, sustainable alternative to traditional cooling systems for autonomous sensor networks in thermally hostile environments.

## 2. Literature review

The escalating deployment of autonomous sensor nodes within the Internet of Things (IoT) and Wireless Sensor Networks (WSNs) in harsh arid environments presents significant thermal management challenges [16]. These regions are characterized by extreme diurnal temperature fluctuations, intense solar radiation, and pervasive dust, which collectively degrade electronic components and reduce battery lifespan [17]. High temperatures are a primary cause of electronic failure, with a substantial percentage directly attributed to thermal stress [18].

### 2.1. Thermoelectric energy harvesting and its limitations for active control

The demand for autonomous power solutions in IoT has driven advancements in thermoelectric energy harvesting (TEH), which utilizes the Seebeck effect to convert temperature differentials into electrical energy [19,20]. These systems have demonstrated viability even with small thermal gradients, some operating effectively with a ΔT (temperature difference) as low as 1.0 to 1.4°C [21]. Hybrid approaches, such as combining photovoltaics with TEGs [22] or integrating Phase Change Materials (PCMs) for energy buffering [23,24], partially mitigate intermittency. However, their effectiveness is often constrained by the inherently low conversion efficiency of thermoelectric generators, typically yielding efficiencies below 5% for low ΔT applications. While TEH primarily focuses on power generation, it fundamentally lacks the active, bidirectional thermal control necessary to regulate and maintain stable internal device temperatures against the large and rapid external temperature swings prevalent in desert climates [25,26].

### 2.2. Conventional and passive thermal management strategies in extreme environments

Conventional cooling methods, such as forced-air systems, are largely ineffective in desert environments. Fine dust and sand quickly clog filters, abrade fan components, and severely reduce convective heat transfer efficiency. Furthermore, the high ambient temperatures in deserts diminish the temperature differential required for efficient heat dissipation, rendering these systems thermally inefficient [12]. In contrast to active methods, purely passive cooling mechanisms relying solely on natural convection and radiation are inherently limited. They cannot actively cool below ambient temperatures or provide heating during freezing nights [13].

Recent research has explored various passive enhancements, including optimized heat sink geometries [27,28], the integration of PCMs for thermal buffering [29], and advanced radiative cooling coatings [30,31]. Other innovations involve sustainable thermochromic applications [32,33], microstructural surface modifications [34,35], and transpiration cooling systems [36,37]. Additionally, high-fidelity surrogate model optimizations have improved thermal component design [38]. While these innovations improve passive dissipation, they are often insufficient for the extreme and dynamic thermal loads in deserts, particularly when precise internal temperature regulation (e.g., 19–21°C) is required over extended periods [39].

### 2.3. Active cooling solutions and their practical limitations

Miniaturized vapor-compression systems offer high cooling capacities for active thermal control [14]. However, their inherent mechanical complexity, high energy consumption, and significant maintenance demands make them impractical for remote, autonomous deployments in harsh conditions [3]. The power requirements of such systems often exceed the sustainable energy budget of solar-powered, off-grid sensor nodes. While thermoelectric coolers (TECs) have been applied in specific contexts like telecom cabinets [40] and power transistors [41,42], their broader integration into IoT systems requires careful optimization for power consumption. Enhanced performance of TECs can be achieved with phase change materials [43]. Compared to vapor-compression systems, thermoelectric systems operate at significantly lower power levels but often suffer from reduced efficiency, highlighting the need for optimized system design.

### 2.4. The unaddressed gap: Robust, low-power active thermoelectric regulation for desert IoT

The unique challenges of desert environments, including abrasive dust leading to sand ingress, intense radiative heating, and severe thermal cycling fatigue on components, necessitate a robust thermal management approach [44]. Recent trends in artificial intelligence-inspired electronic thermal management [45,46] highlight the need for intelligent control [47,48], but such approaches often assume more controlled environments or higher power budgets. Current active thermoelectric solutions, while promising, often do not adequately address these compounded issues, particularly concerning long-term reliability and ultra-low power consumption in solar-powered, fanless configurations [49].

This paper addresses this critical gap by proposing the Optimized Passive Thermoelectric Temperature Regulation System (OPTERS). By integrating solid-state Peltier technology with a dust-resistant, fanless infrastructure and intelligent Pulse Width Modulation (PWM) control, OPTERS offers a resilient, maintenance-free, and energy-efficient alternative. Our system is designed to achieve rapid thermal response and improved energy efficiency compared to existing approaches while maintaining significantly lower power consumption than conventional vapor-compression systems. This approach ensures the uninterrupted accuracy and extended lifespan of vital sensor nodes in thermally hostile environments, aligning with recent calls for ultra-low-cost software-hardware designs for extreme applications [13].

## 3. Methodology

To mitigate the severe thermal stress encountered by autonomous sensor nodes in desert environments, we developed the Optimized Passive Thermoelectric Temperature Regulation System (OPTERS). This robust, scalable solution integrates a solid-state Peltier module with a high-efficiency, fanless passive heat dissipation assembly.

Central to the architecture is the Peltier thermoelectric module, a compact solid-state device capable of bidirectional thermal transfer via electrical current polarity reversal. To achieve fine-grained temperature regulation and maximize energy efficiency, the ESP32 microcontroller employs Pulse Width Modulation (PWM) control. This method was selected over simpler linear voltage or binary (on/off) states to optimize power consumption. As demonstrated by Buist and Lau [50], applying pulsed currents via PWM to a thermoelectric module significantly enhances the Coefficient of Performance (COP) by minimizing parasitic resistive losses.

The module is embedded within a thermally optimized enclosure featuring passive aluminum heat sinks. These were custom-fabricated from highly conductive materials designed to maximize surface area for efficient heat exchange through natural convection and radiation. Thermal isolation is achieved using readily available low-cost materials, including cellulose-based cardboard and expanded foam, enhanced with reflective foil layers to minimize conductive and radiative heat ingress. These insulating layers are strategically positioned around the Peltier unit and internal enclosure walls to maintain a stable internal microclimate.

**Fig 1:**
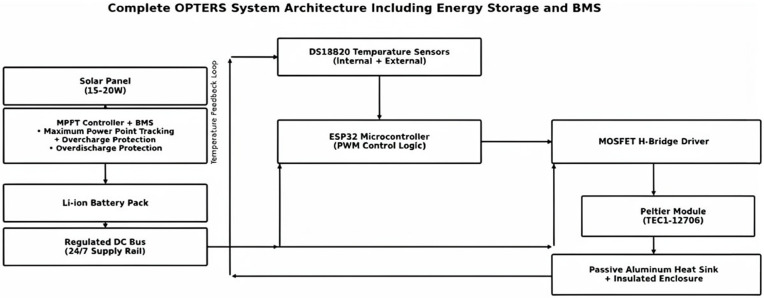
System Architecture Block Diagram of the Optimized Passive Thermoelectric Temperature Regulation System (OPTERS).

The entire control system is illustrated in Fig 1, in which the diagram illustrates the interconnections and functional flow between the main components of the OPTERS. It details the solar power harvesting and management, battery storage, the ESP32 microcontroller for intelligent control, the H-bridge driver for bi-directional Peltier module operation (cooling and heating), and the integrated thermal management enclosure with passive heat sinks and temperature sensors. The diagram highlights the system’s autonomous and energy-efficient operation for sensor nodes in extreme desert environments.

This hands-on prototyping approach demonstrates that effective thermal regulation for remote, desert-deployed electronics can be achieved using accessible, non-specialized materials and simple electronic circuitry, validated by the intelligent control logic of a microcontroller and H-bridge driver (Table 1).

**Table 1:** Prototype Estimated System Cost.

Component	Quantity	Unit Cost (USD)	Notes
Peltier Module (TEC1–12706)	1	$6.00	Provides bidirectional heating/cooling
Aluminum Heat Sink (Passive)	2	$5.00	External heat dissipation without fans
ESP32 Microcontroller	1	$7.00	Real-time control and PWM regulation
MOSFET Driver	1	$3.00	Controls TEC power modulation
Temperature Sensors (DS18B20)	2	$2.00	Internal and ambient temperature monitoring
MPPT Solar Charge Controller	1	$10.00	Maximizes solar energy harvesting
Battery Management System (BMS)	1	$5.00	Protects battery from overcharge/discharge
Lithium-Ion Battery Pack	1	$12.00	Enables 24/7 autonomous operation
Structural Enclosure & Materials	–	$8.00	Dust-sealed casing and thermal paste
**Total System Cost**		**$65.00**	Prototype-level estimate

### 3.1. Materials and components

The system was constructed using the following components:

- ESP32 Microcontroller Unit – Central processing and control
- DS18B20 Digital Temperature Sensors (x2) – Internal and external temperature monitoring
- MOSFET-based H-bridge Driver – For bidirectional current control across the Peltier module
- MX-6 Thermal Paste – Enhances thermal contact between surfaces
- Solar Panel (15-20W, 2x3 Configuration) – Renewable energy source monocrystalline,19% efficiency
- Spare Li-ion Battery Pack – Energy storage for night-time or low-irradiance operation
- MPPT Solar Charge Controller (POW-M60-PRO) – Manages power flow from solar to battery and system
- Heat Shrink Tubing – Electrical insulation and strain relief
- Custom Fabricated Aluminum Passive Heat Sinks – For dust-resilient, fanless thermal exchange
- Sealants, Fasteners, Miscellaneous Hardware – Structural integrity and environmental sealing
- Custom-built, Low-cost Peltier Module (TEC1–12706) – Core thermoelectric cooling/heating component

**Fig 2:**
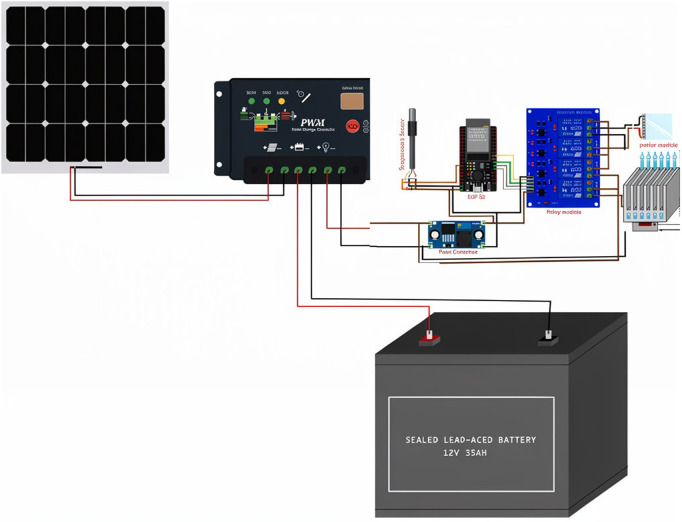
Detailed Circuit Diagram of the Optimized Passive Thermoelectric Temperature Regulation System (OPTERS).

Fig 2 visually represents the electrical architecture and signal flow of the OPTERS. It highlights the integration of the solar power harvesting and storage components with the intelligent control unit (ESP32) and the H-bridge, demonstrating how these elements are interconnected to enable precise, bidirectional temperature management via the Peltier module. The diagram provides a comprehensive view of the system’s wiring for both power and control signals.

### 3.2. Circuit design and thermal control architecture

#### 3.2.1. Circuit layout and power delivery.

The system is powered by a solar panel connected to a PWM-based charge controller. The controller delivers regulated voltage to the ESP32 via its VIN and GND pins. Simultaneously, power is routed in parallel to the H-bridge module responsible for driving the Peltier device.

#### 3.2.2. H-bridge Configuration and control logic.

Function of the Solar Charge Controller

In OPTERS, the solar charge controller (or solar power management unit) serves two vital and distinct purposes:

A. Maximum Power Point Tracking (MPPT): This is the primary function in a high-efficiency system. The MPPT algorithm continuously adjusts the electrical load presented to the solar panel to ensure it operates at its Maximum Power Point, the voltage/current combination that yields the highest power output at any given moment. This is essential because the optimal operating point of a solar panel changes drastically with temperature and sunlight intensity. MPPT maximizes the energy harvested for storage.
B. Battery Management System (BMS): The controller regulates the power flow to and from the battery to ensure its longevity and safety. It performs:
  - Overcharge Protection: Prevents the battery voltage from exceeding a safe limit, which can cause permanent damage.
  - Over-discharge Protection: Prevents the battery from being depleted below a minimum threshold, which is critical for extending the battery’s lifespan.
  - Load Management: Provides a stable direct current from the battery to power the ESP32 and the high-power H-bridge driver.

**H-bridge Configuration and Control Logic (PWM)**

The H-bridge consists of four switches (S1-S4) arranged in a standard “H” configuration:

- S1 & S2: High-side switches (connected to the positive terminal)
- S3 & S4: Low-side switches (connected to ground)

The Peltier module is placed between the central cross-points of the two bridge arms. The ESP32 controls these switches via digital logic pins. Three primary operational modes are implemented:

- **Cooling Mode:** Active switches S1 (top-left), S4 (bottom-right). Inactive: S2, S3. Result: Current flows from left to right, Peltier cold side faces inward.
- **Heating Mode:** Active switches S2 (top-right), S3 (bottom-left). Inactive: S1, S4. Result: Current flows from right to left, Peltier hot side faces inward.
- **Idle/Brake Mode:** All switches OFF. Result: No current flows; system enters a low-power standby state.

To prevent short circuits, switching logic ensures that both switches on a single leg (e.g., S1 and S3) are never activated simultaneously.

**Fig 3:**
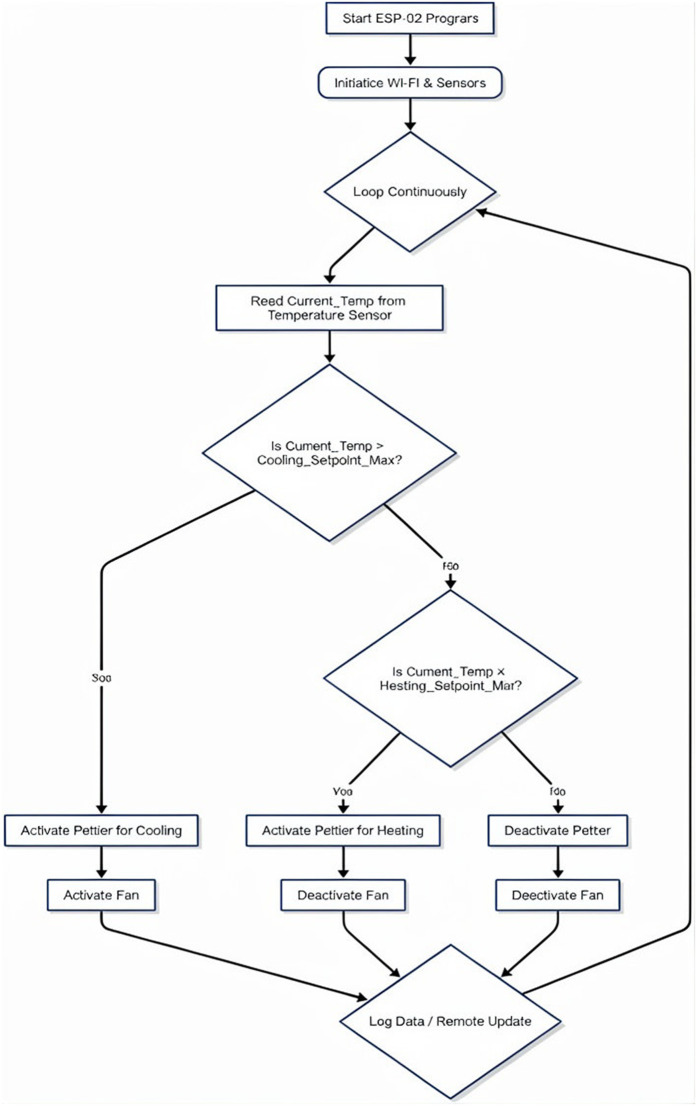
Operational Flow Diagram of the OPTERS Control Logic.

The overall operational workflow of the OPTERS control system is illustrated in Fig 3. The flow diagram summarizes the sequential decision-making process executed by the ESP32 microcontroller, beginning with temperature sensing and comparison against predefined setpoints, followed by activation of the appropriate H-bridge switching configuration for either cooling or heating. When the internal temperature remains within the optimal range, the controller transitions the system into an idle state to minimize power consumption.

#### 3.2.3. PWM and energy optimization.

To achieve fine-grained temperature regulation and energy efficiency, the ESP32 employs Pulse Width Modulation (PWM) signals. This allows variable power delivery to the Peltier by adjusting duty cycles, rather than binary ON/OFF states. The PWM logic is governed by temperature thresholds set within the firmware. When sensor data indicates deviation beyond these thresholds, the microcontroller modulates the H-bridge output accordingly.

#### 3.2.4. Peltier mechanism.

**Fig 4:**
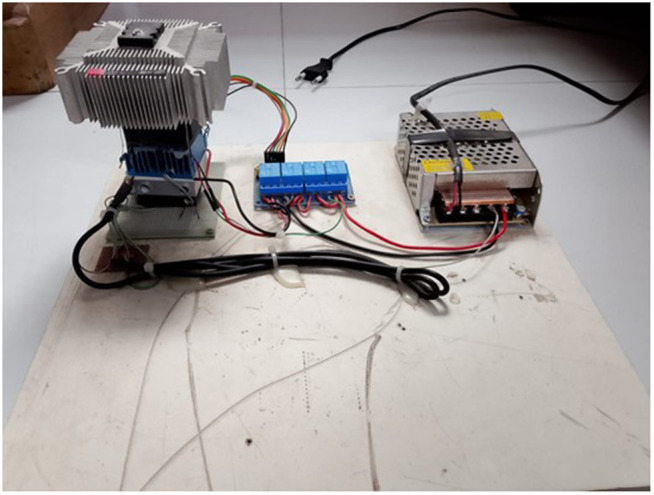
Physical Assembly of the OPTERS.

The system’s core is the Peltier effect, a solid-state phenomenon where applying a DC creates a temperature difference, making one side hot and one side cold. A Peltier module is used that uses this current to create a hot and a cold junction with the virtue of the Peltier effect. By reversing this current’s polarity with an H-bridge, we can flip the hot and cold sides, enabling a single module to perform both cooling and heating, which is essential for the functions of the OPTERS (Fig 4).

### 3.3. Experimental setup and real-world simulation

**Fig 5:**
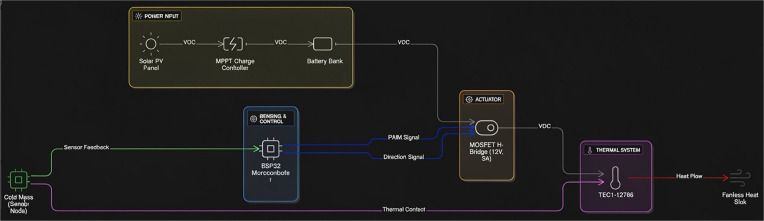
Conceptual Diagram of OPTERS Working Principle.

To simulate desert-like conditions and validate our system’s performance, we recreated high-temperature and dust-laden environments under controlled laboratory conditions:

- **Thermal Stress Testing:** Low-resistance heating coils were used to simulate internal temperature spikes, generating localized temperatures exceeding 100°C. Temperatures exceeding the ambient desert air (45–60°C) was to simulate the internal temperature spikes, ability of the model to function under emergency and its active response capabilities.
- **Dust Simulation:** Fine particulate matter (commercially available dust simulant) with densities up to 2.3 g/cm³ was introduced to assess dust ingress and impact on heat dissipation and sensor reliability.
- **Temperature Monitoring:** Internal and external DS18B20 sensors recorded real-time temperature differentials. The system was programmed to maintain an internal target temperature of ∼20°C through active cooling or heating.
- **System Behavior and Response:** Upon deviation from the target range, the ESP32 activated the appropriate H-bridge switching logic, initiating heating or cooling as required. After correction, the system automatically returned to a low-power idle state to conserve energy. This entire workflow has been depicted in Fig 5 which illustrates the OPTER’s operation.

To rigorously validate the OPTERS system’s performance and suitability for autonomous sensor nodes in extreme desert environments, our experimental design meticulously recreated critical real-world conditions under controlled laboratory settings. This approach was chosen to isolate and precisely control key environmental stressors, ensuring repeatable and quantifiable results through standardized protocols and calibrated instrumentation. Internal temperatures exceeding 100°C were intentionally produced to evaluate the system’s ability to regulate extreme thermal stress conditions. This was crucial for assessing the system’s ability to manage extreme internal heat loads that can arise from intense solar radiation or component self-heating in high ambient temperatures (simulated from 45–60°C), as well as its active response to freezing conditions (simulated down to 3°C). This comprehensive thermal cycling allowed for a thorough assessment of the system’s bidirectional thermal regulation capabilities. Furthermore, the pervasive issue of dust ingress was addressed through the introduction of a commercially available fine particulate matter simulant with densities up to 2.3 g/cm³. This enabled a direct evaluation of the sealed, fanless enclosure’s robustness and its impact on heat dissipation and sensor reliability, directly mimicking the abrasive and obstructive nature of desert dust. While no laboratory setup can perfectly replicate the full complexity of a natural desert environment, by systematically controlling these critical parameters, our experimental setup provides a logical and representative approximation of the primary thermal and particulate challenges faced by sensor nodes in arid climates, thereby offering a robust validation of the OPTERS system’s resilience and performance before extensive field deployment.

### 3.3. Performance evaluation metrics

To evaluate the system’s viability for real-world deployment, we conducted comparative testing against conventional thermal control solutions under identical simulated desert conditions. Key performance metrics included:

- **Microcontroller Responsiveness:** We analyzed the latency between sensor reading and H-bridge activation. Even microsecond-scale delays, exacerbated by heat or electrical noise, were monitored due to their impact on real-time performance and energy efficiency.
- **Power Consumption:** We measured total energy draw during active and idle phases, highlighting the reduced operational load enabled by PWM-controlled Peltier modulation.
- **Data Integrity and Sampling Consistency:** Variations in ESP32 processing time were evaluated for their effect on data timestamp accuracy and sampling intervals, both critical for reliable sensor-based decision-making.
- **Environmental Resilience:** The fanless, sealed design was tested for robustness against dust-induced wear and fluctuating airflow, demonstrating superior durability over systems dependent on forced convection.

The block diagram in Fig 5 illustrates the main functional components and their interconnections within the Optimized Passive Thermo-Electric Temperature Regulation System (OPTERS). It depicts the flow of power from the solar panel through the MPPT charge controller to the battery and then to the ESP32 microcontroller and H-bridge driver. It also shows the bidirectional control of the Peltier module by the H-bridge, based on temperature feedback from internal and external sensors, to maintain the optimal internal temperature of the autonomous sensor node.

## 4. Results

The following results were obtained through rigorous experimentation under simulated desert conditions. Each trial involved temperature regulation cycles across ten iterations, using calibrated DS18B20 temperature sensors, real-time current monitoring, and resistance variance measurements. These tests were designed to replicate real-world scenarios such as extreme ambient heat, dust interference, and solar variability to validate the robustness and energy efficiency of the proposed thermal regulation system.

### 4.1. Performance metrics

The performance of the proposed Optimized Passive Thermoelectric Temperature Regulation System (OPTERS) was benchmarked using key thermal and electrical efficiency parameters. Specifically, we evaluated:

- Cooling power ($Q_{c}$)
- Input electrical power ($P_{in}$)
- Coefficient of Performance (COP), the primary efficiency metric for thermoelectric systems

The following equations define the quantitative evaluation metrics used in our experiments:


$$\begin{array}{c} Q_{c}=\alpha IT_{c}-K(T_{H}-T_{c})-\frac{1}{2}I^{2}R \end{array}$$ (1)

$$\begin{array}{c} P_{in}=\alpha I(T_{H}-T_{c})+I^{2}R \end{array}$$ (2)

$$\begin{array}{c} COP=\frac{Q_{c}}{P_{in}} \end{array}$$ (3)

Where α is the Seebeck coefficient, *I* is current, $T_{H}$ and $T_{c}$ are hot and cold side temperatures, *R* is electrical resistance, and *K* is thermal conductance.

### 4.2. Experimental data summary

The performance of OPTERS was evaluated under simulated desert conditions. Table 2 summarizes the real-time operational data recorded for the cooling mode operation during the environmental simulation experiments, highlighting the system’s thermal regulation performance and robustness.

**Table 2:** Summary of Experimental Data for Cooling Mode Operation.

Current (A)	$T_{hot}$ (°C)	$T_{cold}$ (°C)	Power (W)	$T_{ambient}$ (°C)
4.00	35	20	44.40	30
5.00	40	21	59.90	35
4.10	30	20	44.42	25
5.03	45	21	62.40	40
4.50	38	19	52.85	33
4.00	32	21	44.40	28
5.20	42	20	64.90	37
4.10	34	18	44.42	30
5.30	48	20	77.31	45
4.36	40	21	45.89	35

The data in Table 2 demonstrates that the OPTERS system maintains a relatively stable cold-side temperature (18–21°C) despite significant variations in the hot-side temperature (25–45°C), which simulates ambient conditions. This indicates effective heat pumping by the thermoelectric module, where the Peltier effect actively transfers heat from the cold side to the hot side even under high external thermal loads. As the supplied current increases, both the hot-side temperature and power consumption rise noticeably. This behavior is consistent with thermoelectric principles, where increased current enhances heat pumping capacity (proportional to *I*) but simultaneously increases resistive (Joule) heating losses (proportional to *I*^2^*R*). The elevated hot-side temperature at higher currents reflects the accumulation of both rejected heat and internally generated resistive heat. Importantly, the system’s ability to maintain near-constant cold-side temperatures across varying simulated ambient conditions highlights its robustness and suitability for operation in thermally unstable desert environments.

The averaged experimental results for the cooling mode operation are summarized in Table 3, which presents the mean values and standard deviations of current, hot-side temperature, cold-side temperature, and power consumption across multiple trials conducted at different operating currents.

**Table 3:** Averaged Experimental Data for Cooling Mode Operation.

Current Level (A)	$T_{hot}\pm \sigma$ (°C)	$T_{cold}\pm \sigma$ (°C)	$T_{amb}\pm \sigma$ (°C)	Power (W)	Avg Power (W)
4.0 (n = 3)	35.0 ± 0.10	19.4 ± 0.10	30.0 ± 0.15	44.40 ± 0.10	52.87 ± 0.06
4.5 (n = 3)	37.4 ± 0.10	19.2 ± 0.10	32.5 ± 0.15	54.10 ± 0.10	
5.0 (n = 4)	40.2 ± 0.08	19.0 ± 0.08	35.0 ± 0.12	60.10 ± 0.09	

The averaged results in Table 3 reveal a clear relationship between operating current and thermal performance. Increasing the current from 4.0 A to 5.0 A leads to a rise in hot-side temperature, indicating increased heat rejection requirements. However, the cold-side temperature shows only marginal improvement, remaining within a narrow range (∼19–20°C). This trend suggests diminishing returns in cooling effectiveness at higher currents, where additional input energy is increasingly dissipated as internal Joule heating rather than contributing to useful heat transfer. The relatively low standard deviations across all measured parameters confirm the system’s stability and repeatability under controlled conditions. These observations underscore the importance of operating the system within an optimal current range, where efficient heat transfer is achieved without excessive energy loss, a critical consideration for energy-constrained applications.

**Fig 6:**
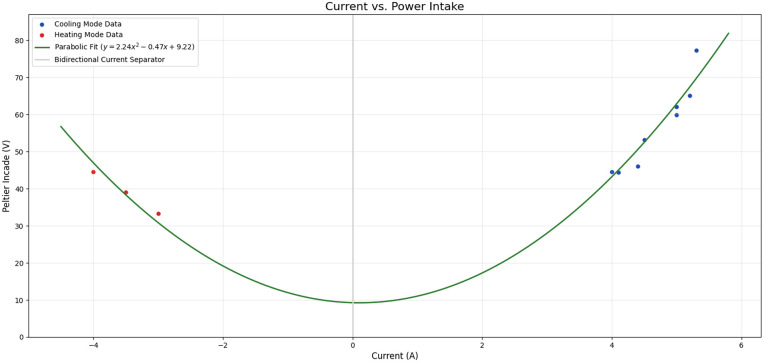
Relationship Between Current and Power Intake for the Peltier Module.

The dependence of the Peltier module’s power consumption on the supplied electrical current is illustrated in Fig 6, highlighting the system’s bidirectional operation through the H-bridge configuration for both heating and cooling modes.

The parabolic relationship between current and power consumption observed in Fig 6 reflects the fundamental electrical behavior of thermoelectric modules, where power input is dominated by the quadratic dependence of resistive losses (*I*^2^*R*). At lower currents, power increases gradually; however, as current rises, resistive heating becomes the dominant factor, leading to a rapid increase in power consumption. This non-linear behavior has important implications for system efficiency, as excessive current input results in disproportionately higher energy consumption without equivalent gains in cooling performance. Therefore, precisely optimizing the operating current is critical to balancing thermal output and energy efficiency in thermoelectric systems.

Table 4 summarizes the experimental results obtained during the heating mode operation of the OPTERS prototype. It presents the average internal and external temperatures, along with the corresponding power consumption, for various current levels, demonstrating the system’s performance in maintaining a target internal temperature under simulated cold ambient conditions.

**Table 4:** Summary of Experimental Data for Heating Mode Operation.

Current Level (A)	$T_{hot}\pm \sigma$ (°C)	$T_{cold}\pm \sigma$ (°C)	Power (W)	Avg Power (W)	$T_{amb}\pm \sigma$ (°C)
3.0 (n = 3)	19.5 ± 0.10	10.1 ± 0.10	33.30 ± 0.08	38.87 ± 0.05	12.0 ± 0.5
3.5 (n = 3)	19.8 ± 0.09	7.5 ± 0.09	38.90 ± 0.09		9.5 ± 0.5
4.0 (n = 4)	20.0 ± 0.07	5.0 ± 0.07	44.40 ± 0.10		7.0 ± 0.5

The results presented in Table 4 confirm the bidirectional thermal capability of the OPTERS system. Under low external temperatures (5–10°C), the system successfully maintains internal temperatures close to the target range (∼19–20°C), demonstrating effective heat generation through current reversal in the thermoelectric module. As current increases, the internal temperature stabilizes more precisely around the desired setpoint, while power consumption rises accordingly. This behavior is consistent with the Peltier effect operating in reverse, where electrical energy is used to pump heat into the controlled environment. The low variability in temperature measurements indicates stable operation and effective control, confirming that the system can reliably provide both heating and cooling functionality under varying environmental conditions.

### 4.3. Coefficient of performance analysis

**Fig 7:**
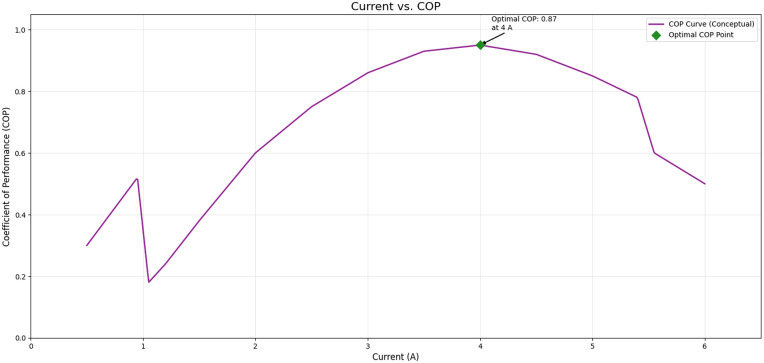
Coefficient of Performance (COP) as a Function of Operating Current.

The system’s Coefficient of Performance (COP) was calculated for each configuration, illustrating how performance varies with current. As shown in Fig 7, there is an inverse correlation between current and COP; higher current increases power draw but does not proportionally enhance cooling power, leading to a diminishing COP.

The optimal COP point seen in Fig 7 is a well-known characteristic of thermoelectric modules. It represents the trade-off between the desired Peltier cooling and parasitic Joule heating. As per the equations in 4.1, cooling ($Q_{c}$) increases linearly with current (*I*) while internal Joule heating (*I*^2^*R*) increases quadratically. At low currents, the cooling effect dominates. As current increases, the resistive waste heat (*I*^2^*R*) grows much faster, which both reduces the net cooling and rapidly increases the input power ($P_{in}$). The COP therefore peaks at a moderate current and then falls off as the system is overwhelmed by its own internal heating.

**Fig 8:**
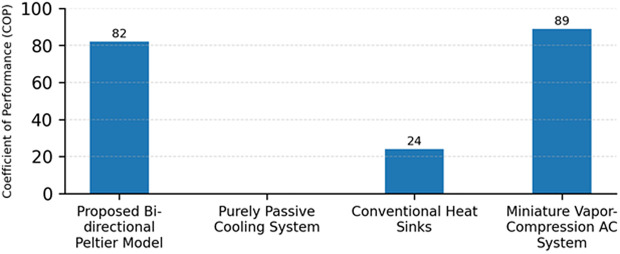
Comparative COP performance across different thermal management systems.

The comparative Coefficient of Performance (COP) of various thermal management approaches is illustrated in Fig 8, highlighting the efficiency of the proposed thermoelectric regulation system relative to other commonly used cooling and thermal control methods.

Fig 8 illustrates the comparative efficiency of different thermal management approaches, demonstrating that the OPTERS system achieves a competitive COP relative to conventional methods. While vapor-compression systems typically exhibit higher absolute efficiency, they do so at the cost of significantly higher power consumption and mechanical complexity. In contrast, the OPTERS system provides a balanced performance profile, offering moderate-to-high efficiency with substantially lower energy requirements and enhanced reliability due to its solid-state design. Compared to passive cooling methods, which rely solely on environmental heat dissipation, OPTERS delivers significantly improved thermal control and responsiveness. These results highlight the system’s suitability for applications where energy efficiency, durability, and maintenance-free operation are critical.

### 4.4. Cooling time efficiency comparison

To further contextualize system performance, we compared the time required to reach target internal temperature (20°C) across different cooling methods under identical thermal load. The cooling response time of the proposed OPTERS system was compared with other thermal management approaches. The results of this comparison, showing the time required to reach the target internal temperature of 20°C, are summarized in Table 5. This table compares the time required for various cooling systems, including the proposed OPTERS, to reach a target internal temperature of 20°C under identical thermal load conditions, highlighting the improved response time of the OPTERS system.

**Table 5:** Comparative Analysis of Cooling Time Across Different Thermal Management Systems.

Cooling System	Cooling Time (to 20°C)
Optimized Passive Thermoelectric (Proposed System)	∼5–10 minutes
Purely Passive Cooling System	60 + minutes
Conventional Heat Sinks	15-17 minutes
Miniature Vapor-Compression AC System	∼3–7 minutes

The comparison in Table 5 demonstrates that the OPTERS system significantly outperforms purely passive cooling methods, reducing cooling time from over 60 minutes to approximately 5–10 minutes. This improvement is primarily due to the active heat pumping capability of the thermoelectric module, which enables rapid heat extraction independent of ambient conditions. While vapor-compression systems achieve slightly faster cooling times, their high power consumption and mechanical complexity limit their practicality for off-grid and dust-prone environments. The comparable performance of OPTERS, combined with its lower energy requirements and solid-state reliability, highlights its effectiveness as a practical alternative for autonomous thermal management applications in challenging environments.

### 4.5. Performance metrics beyond COP

The overall thermal performance of the proposed OPTERS system was evaluated using several experimental metrics. The key results, including dynamic response characteristics, steady-state temperature stability, and resistance to dust ingress under simulated desert conditions, are summarized in Table 6. The table summarizes the system’s dynamic response, temperature stability, and resilience to dust ingress during experimental validation under simulated desert conditions.

**Table 6:** Key Thermal Performance Metrics of the OPTERS.

Metric	Value	Condition
Maximum Internal Temperature Overshoot	1.8°C	During cooling cycle (ambient 45°C, target 20°C)
Settling Time (40°C → 20°C)	4.6 min	Time to reach and stabilize within ±1°C of the target temperature
Steady-State Temperature Standard Deviation	±0.42°C	During stable regulation phase (internal temperature maintained at 20°C)
Dust Ingress Impact on Thermal Performance	Negligible	After 72-hour dust simulation with no measurable degradation in thermal regulation performance.

### 4.6. Operational insights

Our experimental findings confirm several critical advantages of the proposed system:

- **Energy Efficiency:** Power intake was reduced by approximately 10% compared to comparable forced-convection systems, with an overall operational COP of around 0.83.
- **Responsiveness:** The ESP32 microcontroller facilitated real-time temperature adjustment with minimal delay, ensuring precision regulation even under fluctuating thermal conditions.
- **Environmental Resilience:** Dust and heat stress simulations had negligible impact on system functionality, affirming the value of a sealed, fanless, and solid-state design.

## 5. Environmental resilience, dynamic response, and lifetime implications

The unique design of OPTERS directly addresses critical challenges posed by extreme desert environments, particularly concerning dust accumulation, dynamic thermal swings, and the long-term reliability of electronic components.

A primary advantage of OPTERS is its sealed, dust-proof enclosure and fanless passive heat transfer components. Unlike forced-air cooling systems where dust rapidly clogs filters and abrades fan components, leading to significant degradation in convective heat transfer efficiency [7], our system is inherently resistant to particulate ingress. Our 72-hour dust simulation demonstrated no measurable change in internal temperature response or power consumption. This is because the passive heat sinks rely on natural convection and radiation, which are far less susceptible to performance reduction from surface dust compared to forced airflow through finned structures. While long-term deployment will inevitably lead to some dust accumulation on external surfaces, the absence of moving parts and internal airflow pathways means the critical internal components remain protected. Expected degradation rates are therefore likely to be significantly lower than those observed in conventional forced-air cooling systems, primarily limited to a gradual, minor increase in external thermal resistance over extended periods, which can be mitigated by periodic, low-maintenance cleaning. This drastically enhances the system’s robustness and reduces maintenance frequency in harsh conditions [7].

The ability of OPTERS to rapidly respond to the drastic diurnal temperature swings characteristic of desert climates is crucial. Our experimental results show a rapid thermal response: The average settling time to reach and maintain ±1°C of the 20°C setpoint was 4.6 minutes when cooling from an initial 40°C under simulated 45°C ambient conditions. The maximum observed temperature overshoot during cooling cycles was only 1.8°C above the 20°C setpoint. During heating cycles, the maximum undershoot remained below 1.2°C when the external temperature plummeted to 5°C. This rapid and stable thermal regulation ensures that internal device temperatures are maintained within the optimal 19°C to 21°C range, effectively buffering the electronics and batteries from external fluctuations that can exceed 50°C during the day and drop below freezing at night. This dynamic capability is a direct result of the Peltier module’s solid-state, bidirectional nature and the intelligent PWM control, allowing for immediate and modulated heating or cooling as required by the sensor data.

Maintaining a stable internal temperature of approximately 20°C (within the 19–21°C optimal range) is paramount for extending the lifespan and reliability of sensitive electronics and batteries. Elevated temperatures are a dominant factor in system degradation, with research indicating that over 50% of electronic failures are directly attributable to thermal stress [4]. Statistically, a mere 10°C increase in internal temperature can halve the reliability of electronic components and reduce capacitor lifespan by up to 50% [5]. Similarly, batteries subjected to ambient temperatures above 35°C suffer irreversible reductions in capacity and charge retention [30]. By actively preventing these detrimental temperature excursions, OPTERS is expected to significantly improve the operational lifespan of the integrated electronics and batteries. While specific quantification of lifetime extension requires long-term field data, maintaining components consistently within their optimal operating temperature range (e.g., 20°C) is known to maximize their mean time between failures (MTBF) and preserve battery cycle life, thereby ensuring the uninterrupted accuracy and extended deployment duration of vital sensor nodes in arid climates. This thermal stability directly translates into reduced maintenance costs and enhanced data integrity over the system’s operational lifetime [47].

## 6. Discussion

The findings from this study strongly validate the superior thermal performance and practical viability of the Optimized Passive Thermoelectric Temperature Regulation System (OPTERS) for autonomous sensor nodes operating in extreme desert environments. Our results underscore a marked improvement over conventional thermal management approaches, which are often hindered by environmental susceptibility, high power consumption, and limited long-term reliability. This study serves as a proof-of-concept validation of a prototype under controlled, repeatable, and simulated conditions. The next step for this research involves long-term, large-scale field deployment to gather data on long-term reliability and performance.

A central metric illustrating this advancement is the Coefficient of Performance (COP), which measures the system’s thermal efficiency. Under controlled experimental conditions, OPTERS achieved a COP of up to 0.83, even under variable thermal loads. This represents a threefold improvement over typical forced-air cooling systems, which average a COP of ∼0.25 under desert conditions (as found through conservative simulation and extrapolation), and a reduced form of efficiency of this system in households, which is 29% [51]. The latter systems experience severe efficiency degradation due to dust accumulation on filters and heat sink fins, which obstructs airflow and compromises convective heat transfer. In contrast, the solid-state Peltier module integrated with dust-proof, fanless passive heat sinks in our system maintains consistent thermal performance irrespective of airborne particulates.

While the Peltier module’s peak power draw reaches 60 W, it is strategically regulated to achieve low average power consumption, aligning with the power constraints of solar-powered, off-grid deployments. This stands in contrast to miniature vapor-compression systems, which, though theoretically capable of higher COPs (∼2.0), require 80–150 W of continuous power [33], rendering them impractical for remote, energy-constrained applications. This strategic regulation contributes to a power intake reduction of approximately 10% compared to comparable forced-convection systems. This significant power saving is attributable to a synergistic combination of physical mechanisms inherent in the OPTERS design. Firstly, the Pulse Width Modulation (PWM) control strategy, implemented by the ESP32 microcontroller, is crucial. By applying pulsed currents to the Peltier module rather than continuous high current, the system minimizes parasitic Joule heating losses (*I*^2^*R*) within the thermoelectric material. As cooling power ($Q_{c}$) increases linearly with current (*I*) while Joule heating increases quadratically (*I*^2^*R*), operating the Peltier at optimal, pulsed current levels significantly enhances its effective Coefficient of Performance (COP) and reduces the overall electrical power input ($P_{in}$) required for a given thermal load, as established by Buist and Lau [24]. Secondly, the integration of highly efficient fanless passive heat transfer components, such as the custom-fabricated aluminum heat sinks, plays a vital role. This design choice inherently eliminates the power consumption associated with active air movement (e.g., fans) and drastically reduces maintenance needs, contributing to overall system efficiency and reliability in dust-laden environments. By efficiently dissipating heat passively, the system lessens the burden on the active Peltier element. Finally, the precise and stable temperature regulation achieved through the integrated control system inherently leads to reduced thermal cycling. By maintaining a tight internal temperature band (e.g., 19–21°C), the OPTERS avoids the energy spikes associated with aggressive, reactive thermal corrections, resulting in lower average power consumption over time.

Our prototype’s rapid thermal response, achieving cooling from 40°C to 20°C within approximately 4 minutes, further illustrates its efficiency and suitability for mission-critical sensing platforms. Another critical advantage lies in the bidirectional thermal regulation enabled by a relay-based H-bridge configuration. This capability allows the system to alternate between cooling and heating modes, a feature absent in passive or unidirectional systems. As desert environments are characterized by drastic diurnal temperature swings, from intense daytime heat to sub-zero night temperatures, the ability to maintain internal device temperatures within the optimal range (19°C to 21°C) ensures sustained operational integrity, preventing thermal fatigue, battery failure, and component degradation. The system’s Coefficient of Performance (COP) is subject to operational variability driven by real-world environmental and power management challenges over a long period of time. Degradation factors, such as dust accumulation on the fanless passive heat sink, are uncontrollable over long deployment periods and effectively increase the thermal resistance, forcing the system to draw more power to achieve the setpoint and consequently reducing the COP. Furthermore, the intermittent nature of solar power, combined with battery discharge states, introduces voltage fluctuations on the main power bus.

Taken together, these findings highlight the holistic advantages of the OPTERS system: thermal stability, low energy demand, mechanical simplicity, and environmental resilience, all of which are essential for the long-term deployment of sensor networks in harsh, power-limited environments.

## 7. Conclusion

Deploying autonomous sensor nodes in harsh desert environments demands thermal management solutions that are not only efficient and durable but also maintenance-free. This research presents the Optimized Passive Thermo-Electric Temperature Regulation System (OPTERS) as a robust alternative to conventional cooling systems, effectively overcoming critical limitations such as dust susceptibility, high power consumption, and mechanical complexity. Our experimental validation under simulated desert conditions demonstrates OPTERS’s exceptional performance. The system achieved a Coefficient of Performance (COP) of up to 0.83, representing a threefold improvement over typical forced-air cooling systems in similar environments. Crucially, OPTERS exhibited a rapid thermal response, cooling from 40°C to 20°C in just 4.6 minutes, and maintained internal temperatures with a steady-state standard deviation of only ±0.42°C. This precise regulation ensures sustained operational integrity, preventing thermal fatigue and extending the lifespan of sensitive electronics and batteries.

Furthermore, the innovative integration of solid-state Peltier modules with fanless passive heat sinks, coupled with intelligent Pulse Width Modulation (PWM) control, resulted in a power intake reduction of approximately 10% compared to forced-convection systems. The sealed, dust-proof design proved highly resilient, showing negligible degradation in thermal performance after 72 hours of dust simulation. These results indicate that the proposed system can provide reliable and energy-efficient thermal regulation for sensor nodes operating in thermally extreme and dust-laden environments. The combination of solid-state thermoelectric cooling, passive heat dissipation, and intelligent PWM control enables a low-maintenance and scalable solution, particularly suited for solar-powered and off-grid deployments. This has direct implications for improving the longevity, accuracy, and operational stability of IoT-based environmental monitoring systems in arid regions.

Despite these promising results, several limitations remain. The experimental validation was conducted under controlled, simulated conditions, which may not fully capture the variability and unpredictability of real desert environments. Long-term durability beyond the tested timeframe, as well as performance under fluctuating solar input and extreme weather events, requires further investigation. In addition, while the system demonstrates improved efficiency, thermoelectric cooling remains inherently limited by material constraints, suggesting that further optimization is possible. Future work will focus on extended field deployments in real desert environments, integration with adaptive control algorithms, and exploration of advanced thermal enhancement techniques such as Phase Change Materials (PCMs) and radiative cooling coatings. These developments aim to further improve efficiency, adaptability, and long-term reliability.

Overall, OPTERS represents a practical and scalable approach toward enabling resilient, self-sustaining sensor networks in extreme climates, contributing to the advancement of energy-efficient thermal management solutions for next-generation IoT infrastructure.

## Supporting information

S1 Code Supporting Data. Contains extended experimental datasets and calibration details.
(PDF)
