# Supplementary material for: Optimized passive thermoelectric temperature regulation for dust-resilient sensor nodes in Extreme Desert Climates
Source: PLoS One. 2026 Jun 26;21(6):e0352185. doi: 10.1371/journal.pone.0352185 (PMC13308843; doi:10.1371/journal.pone.0352185)
Supplement: S1 Code — Contains extended experimental datasets and calibration details. (PDF) [file pone.0352185.s001.pdf]

Code:

```
#include <EEPROM.h>
#include <OneWire.h>
#include <DallasTemperature.h>

#define EEPROM_SIZE 512
#define ONE_WIRE_BUS 4  // DS18B20 data pin

#define Pel_Pow_Relay 25  // Relay control pin
#define RELAY_PIN2 27
#define RELAY_PIN3 33
#define OFF 1
#define ON 0

OneWire oneWire(ONE_WIRE_BUS);
DallasTemperature sensors(&oneWire);

void setup() {
  Serial.begin(115200);
  EEPROM.begin(EEPROM_SIZE);
  sensors.begin();

  pinMode(Pel_Pow_Relay, OUTPUT);
  pinMode(RELAY_PIN2, OUTPUT);
  pinMode(RELAY_PIN3, OUTPUT);

  digitalWrite(Pel_Pow_Relay, OFF);

  randomSeed(analogRead(0));
}

void loop() {
  sensors.requestTemperatures();
  float temperature = sensors.getTempCByIndex(0);

  Serial.print("Current Temp: ");
  Serial.print(temperature);
  Serial.println(" °C");

  if (temperature <= 18.0) {
    digitalWrite(RELAY_PIN2, ON);
    digitalWrite(RELAY_PIN3, ON);
    digitalWrite(Pel_Pow_Relay, ON);
```

```
}
else if (temperature >= 22.0) {
  digitalWrite(Pel_Pow_Relay, ON);
  digitalWrite(RELAY_PIN2, OFF);
  digitalWrite(RELAY_PIN3, OFF);
  Serial.println("Coolling");
}
else if (temperature <= 20.0) {
  digitalWrite(Pel_Pow_Relay, OFF);
  delay(3000);
  digitalWrite(RELAY_PIN2, OFF);
  digitalWrite(RELAY_PIN3, OFF);
}

Serial.println("");
Serial.println("");
Serial.println("");
Serial.println("Start Processing");

unsigned long startTime = millis();

// ==== EEPROM Write Random Values ====
for (int i = 0; i < EEPROM_SIZE; i++) {
  byte value = random(0, 256);
  EEPROM.write(i, value);
}
EEPROM.commit();

// ==== Heavy Processing ====
float dummy = 0;
for (unsigned long i = 0; i < 1000000; i++) {
  dummy += sin(i * 0.001) * cos(i * 0.002);
}

// ==== EEPROM Clear ====
for (int i = 0; i < EEPROM_SIZE; i++) {
  EEPROM.write(i, 0);
}
EEPROM.commit();

unsigned long endTime = millis();

Serial.println("End Processing");
Serial.print("Total Processing Time: ");
```

```
  Serial.print(endTime - startTime);
  Serial.println(" ms");
  Serial.println("");
  Serial.println("");

  delay(2000);  // Delay before next loop
}
```

Description

This prototype implements a **thermoelectric temperature management controller** using an **ESP32 (or compatible Arduino-IDE board)** and a **DS18B20 temperature sensor**. The system monitors internal temperature and controls relay outputs connected to a **thermoelectric cooling module (Peltier system)** and related circuitry.

The controller also performs **EEPROM stress testing and computational load simulation** to measure processing time and demonstrate system performance during operation.

The main goal of the program is to:

- Monitor internal temperature
- Control thermoelectric cooling hardware via relays
- Maintain temperature within a defined range
- Simulate processing workload
- Measure processing performance

Hardware Components

The system uses the following hardware components:

- **ESP32 microcontroller**
- **DS18B20 digital temperature sensor**
- **Relay module**
- **Thermoelectric cooling module (Peltier device)**
- **EEPROM (internal flash emulation)**

Pin Configuration

Component	Pin
DS18B20 Data Pin	GPIO 4
Peltier Power Relay	GPIO 25
Relay Output 2	GPIO 27
Relay Output 3	GPIO 33

Constants used in the code:

OFF = 1
ON = 0

These values correspond to **active-low relay modules**, where LOW activates the relay.

Libraries Used

EEPROM Library

Handles reading and writing to the ESP32's flash-based EEPROM.

OneWire Library

Used for communication with the DS18B20 sensor over the OneWire protocol.

DallasTemperature Library

Simplifies temperature measurement from the DS18B20 sensor.

Program Workflow

The program runs in a continuous loop performing the following tasks:

1. **Read Temperature**
2. **Control Thermoelectric System**
3. **Simulate Processing Load**
4. **Write and Clear EEPROM**
5. **Measure Processing Time**

Temperature Monitoring

The DS18B20 sensor measures the current temperature.

Example output in the serial monitor:

Current Temp: 21.50 °C

Temperature is retrieved using:

```
sensors.requestTemperatures();
float temperature = sensors.getTempCByIndex(0);
```

Temperature Control Logic

The system controls relays depending on temperature thresholds.

Cooling Mode

Activated when temperature rises above **22°C**

Actions performed:

- Turn ON Peltier power relay
- Disable RELAY_PIN2 and RELAY_PIN3
- Print status message

Coolling

Low Temperature Mode

Activated when temperature drops below **18°C**

Actions performed:

- Enable RELAY_PIN2
- Enable RELAY_PIN3
- Turn ON Peltier power relay

Stabilization Mode

Activated when temperature drops below **20°C**

Actions performed:

- Turn OFF Peltier relay
- Wait 3 seconds
- Turn OFF auxiliary relays

This helps prevent unnecessary cooling once the target temperature is reached.

EEPROM Processing Test

The system writes **random values to the entire EEPROM memory**, simulating heavy data operations.

```
for (int i = 0; i < EEPROM_SIZE; i++)
{
  byte value = random(0,256);
  EEPROM.write(i,value);
}
```

After writing the data, the program commits the changes:

```
EEPROM.commit();
```

Then it **clears the EEPROM** by writing zeros.

This simulates memory usage during operation.

Computational Load Simulation

The program performs a heavy mathematical loop to simulate processing load.

```
for (unsigned long i = 0; i < 1000000; i++)
{
  dummy += sin(i * 0.001) * cos(i * 0.002);
}
```

This represents computational tasks such as:

- sensor processing
- control algorithms
- communication tasks

Processing Time Measurement

The system measures total processing time using the `millis()` function.

unsigned long startTime = millis();

After completing all operations:

unsigned long endTime = millis();

The difference is printed:

Total Processing Time: XXXX ms

Example output:

Start Processing
End Processing
Total Processing Time: 842 ms

Serial Monitor Output Example

Typical output in the Arduino Serial Monitor:

Current Temp: 23.10 °C

Coolling

Start Processing
End Processing
Total Processing Time: 845 ms

System Behavior Summary

Temperature	System Action
≥ 22°C	Cooling mode activated
≤ 18°C	Low temperature control mode
≤ 20°C	Cooling stops

Delay and Loop Timing

After completing one full cycle, the program waits:

```
delay(2000);
```

This provides a **2-second pause before repeating the loop**.

Notes

- The relay logic assumes **active-low relay modules**.
- EEPROM size is configured as **512 bytes**.
- The program is compatible with **ESP32 Arduino framework**.
- Processing load simulation is included for testing purposes.

License

This project is intended for **educational and research use**, particularly for thermoelectric thermal management systems in embedded environments.
