# Supplementary material for: Optimized passive thermoelectric temperature regulation for dust-resilient sensor nodes in Extreme Desert Climates
Source: PLoS One. 2026 Jun 26;21(6):e0352185. doi: 10.1371/journal.pone.0352185 (PMC13308843; doi:10.1371/journal.pone.0352185)
Supplement: Attachment — Submitted filename: Reviewer Comments PLOS One.pdf [file pone.0352185.s002.pdf]

Response to Reviewers

Manuscript ID: PONE-D-26-06689

Title: Optimized Passive Thermoelectric Temperature Regulation for Dust-Resilient Sensor Nodes in Extreme Desert Climates

We thank the Academic Editor and the Reviewers for their time, constructive feedback, and valuable insights. We have carefully revised the manuscript to address all concerns. Below is our point-by-point response.

Response to Journal Requirements

1 & 3. PLOS ONE Style and LaTeX Template: We have updated the entire manuscript to use the official PLOS ONE LaTeX template and ensured all formatting complies with the journal's style guidelines. 2. Code Sharing: Our system utilizes standard PWM control logic on an ESP32 microcontroller. All algorithmic flow is documented in Figure 3. 4. Preprint/Dual Publication: This has been addressed in our Cover Letter. The preprint is an early, non-peer-reviewed version of this work. 5. Data Availability: We confirm that all raw data required to replicate the results are fully included within the manuscript text and tables (specifically Tables 2, 3, 4, 5, and 6). 6 & 8. Figure and Table Citations: Due to the addition of new diagrams and tables, the numbering has changed. We have meticulously verified that all current figures (Figures 1 through 8) and tables (Tables 1 through 6) are explicitly cited in the text. 7. Figure 3 Copyright: We have completely removed the copyrighted image and replaced it with an original, author-generated diagram that complies with the CC BY 4.0 license.

Response to Academic Editor

Comment: *The readability of this manuscript is very poor. It lacks detailed descriptions of the system design and in-depth analyses of the validation results. Please supplement performance metrics other than COP.*
Response: We have thoroughly rewritten the manuscript to elevate the scientific tone and readability. To provide detailed descriptions of the system design, we added a new System Architecture Block Diagram (Figure 1) and a fully detailed Circuit Diagram (Figure 2). To supplement performance metrics beyond COP, we added Table 6, which now includes maximum temperature overshoot (1.8°C), undershoot (1.2°C), settling time (4.6 min), steady-state temperature standard deviation (±0.42°C), and dust ingress impact.

Response to Reviewer 1

Comment: *Lacks overall presentation and scientific writing. The manuscript needs thorough revision.*

Response: The entire manuscript has been heavily proofread and revised to improve grammar, flow, and scientific rigor.

Comment: *The figure captions need more description. The table titles need revision.*
Response: We have expanded all figure captions and table titles to be fully descriptive and self-contained so readers can understand them without searching the main text.

Comment: *Some figures need to replot with better quality eg. Fig 7.*
Response: We replotted the COP curve (previously Figure 7) to ensure high resolution, clear labeling, and publication-ready quality.

Response to Reviewer 2

Comment: *The current title does not fully reflect the manuscript's scope... Suggested Alternatives...*
Response: We agree. We updated the title to "Optimized Passive Thermoelectric Temperature Regulation for Dust-Resilient Sensor Nodes in Extreme Desert Climates" to better reflect the engineering scope and environment.

Comment: *The manuscript cites some foundational work but lacks engagement with recent thermoelectric, desert environment, and IoT thermal management literature (2020–2024).*
Response: We have heavily revised Section 2 (Literature Review) to incorporate recent literature from 2020 to 2024. We added discussions on hybrid cooling, PCM integration, radiative cooling coatings, and low-power thermoelectric systems.

Comment: *What is the physical mechanism behind the observed 10% reduction in power consumption?*
Response: We expanded Section 6 (Discussion) to clarify this. The reduction is driven by a combination of PWM efficiency minimizing parasitic Joule heating, the zero-power nature of our fanless passive heat sinks, and tight thermal regulation preventing high-power corrective spikes.

Comment: *How does dust accumulation affect thermal performance over time? What is the expected lifetime improvement? What is the thermal response time?*
Response: We created a brand new section, "Section 5: Environmental Resilience, Dynamic Response, and Lifetime Implications", to address these exact questions. We also quantified the rapid thermal response time (4.6 minutes to reach 20°C) and the negligible impact of dust after our 72-hour simulation in Table 6.

Comment: *Avoid general statements such as "superior solution" unless supported by comparative data. Include limitations... Strengthen the conclusion…*
Response: We removed subjective phrases like "superior solution". We also rewrote the Conclusion to clearly state our quantitative findings, practical implications, and explicit limitations (e.g., prototype scale, need for long-term outdoor validation).

Comment: *Provide a cost breakdown to support the "low cost" claim. The methodology section should include a block diagram summarizing system architecture. Ensure consistent*

use of units.
Response: We added a detailed cost breakdown in Table 1, showing the $65 total cost. We added the requested System Architecture Block Diagram as Figure 1. We also standardized all units (°C, W) throughout the text.

Response to Reviewer 3

Comment: *Ambient Temperature Data: The primary concern is the absence of Ambient Temperature (Environment Temperature) in Tables 1.1 and 1.2.*
Response: We have added a dedicated "Ambient Temperature" column to our cooling data tables (now labeled Table 2 and Table 3) to properly demonstrate the thermal gradient.

Comment: *Circuit Schematic: Figure 1 (Circuit Diagram) currently omits the battery and BMS connections. For a system claiming "Autonomous 24/7 operation," a complete schematic including the energy storage interface is necessary.*
Response: Thank you for pointing this out. We completely redrew the schematic (now Figure 2) to explicitly include the Li-ion battery pack and the MPPT solar charge controller, which handles the BMS functionality.
