# Supplementary material for: Optimized passive thermoelectric temperature regulation for dust-resilient sensor nodes in Extreme Desert Climates
Source: PLoS One. 2026 Jun 26;21(6):e0352185. doi: 10.1371/journal.pone.0352185 (PMC13308843; doi:10.1371/journal.pone.0352185)
Supplement: Attachment — Submitted filename: __Response to the reviewers.docx [file pone.0352185.s003.docx]

**Response to Academic Editor**

**Comment**: More explanations are required regarding the rationality of the experimental validation design.

- Our Response: We thank the Academic Editor for highlighting the need for further clarification on the rationality of our experimental validation design. We have incorporated a new paragraph into Section 3.3, "Experimental Setup and Real-World Simulation," to explicitly address this point. The added text provides a detailed explanation of why our laboratory-based simulation approach is logical and how it accurately mimics real desert conditions.
  The new paragraph emphasizes:
  - Controlled Environment: The rationale for using controlled laboratory settings to isolate and precisely manage key environmental stressors, ensuring repeatable and quantifiable results.
  - Simulation of Diurnal Temperature Swings: How low-resistance heating coils were used to generate internal temperature spikes (exceeding 100°C, simulating internal heat loads from solar radiation or component self-heating) and how ambient temperatures were varied (from 3°C to 45-60°C) to comprehensively assess bidirectional thermal regulation.
  - Dust Ingress Simulation: The use of a commercially available fine particulate matter simulant (with densities up to 2.3 g/cm³) to directly evaluate the dust-resistant enclosure and its impact on heat dissipation and sensor reliability.
  - Logical Representation: A concluding statement affirming that, despite the inherent limitations of any laboratory simulation, our systematic control of these parameters provides a logical and accurate representation of the primary thermal and particulate challenges in arid climates, thus robustly validating the OPTERS system's performance.

We believe this expanded explanation thoroughly addresses the editor's comment and provides a stronger foundation for the experimental results presented in the manuscript.

**Response to Reviewer #3**

We thank Reviewer #3 for their insightful comments and constructive feedback, which have significantly helped us improve the clarity and technical robustness of our manuscript. We are pleased to hear that the revised version is suitable for publication according to the other two reviewers. We have carefully addressed the points raised.

Reviewer Comment 1: Completeness of the Technical Schematic (Figure 1)

- Original Comment: "The system is described as capable of 'Autonomous 24/7 operation' through solar harvesting and battery storage. However, the circuit diagram in Figure 1 currently omits the battery pack and its interface. To serve as a robust technical reference, the schematic must be updated to include the actual wiring for the battery, MPPT charge controller, and Battery Management System (BMS) mentioned in the methodology."
- Our Response: We appreciate the reviewer's keen observation regarding the completeness of Figure 1. We agree that a comprehensive technical schematic is crucial for a robust technical reference, especially given the system's autonomous 24/7 operation capabilities. We confirm that the updated Figure 1 (now Figure 2 in the revised manuscript, as Figure 1 is now the system architecture block diagram) has been revised to include the detailed wiring for the solar panel, MPPT solar charge controller, Li-ion battery pack, and the Battery Management System (BMS). This updated schematic now clearly illustrates the electrical connections and control pathways for power harvesting, storage, and distribution, providing a more complete and accurate representation of the OPTERS system's power management architecture. We believe this enhancement significantly improves the technical clarity and utility of the figure for readers.

Reviewer Comment 2: Inclusion of Ambient Temperature Data in Tables 1.1 & 1.2

- Original Comment: "Currently, Tables 1.1 and 1.2 list temperatures for the hot and cold sides of the module but do not include the Ambient (Environmental) Temperature for each recorded data point in cooling mode. Providing the external temperature baseline for every experiment is essential to demonstrate the actual thermal gradient achieved and to verify the reported Coefficient of Performance (COP)."
  - Our Response: We sincerely thank the reviewer for highlighting this critical point. We completely agree that the external temperature baseline is essential to demonstrate the actual thermal gradient and validate the COP under simulated desert conditions. Please note that during the revision process, the original Tables 1.1 and 1.2 were renamed and reformatted as Table 2 and Table 3 in the current manuscript. As requested, we have ensured that the Ambient (Environmental) Temperature corresponding to every recorded data point is explicitly included as a dedicated column in both Table 2 (real-time data) and Table 3 (averaged data). We hope this fully clarifies the thermal gradients achieved during our experimental validation and satisfies the requirement for reproducibility.
