# Supplementary material for: Optimized passive thermoelectric temperature regulation for dust-resilient sensor nodes in Extreme Desert Climates
Source: PLoS One. 2026 Jun 26;21(6):e0352185. doi: 10.1371/journal.pone.0352185 (PMC13308843; doi:10.1371/journal.pone.0352185)
Supplement: Peer review history

# Peer review: Decision Letter 0

**DOI:** 10.1371/journal.pone.0352185.r001

**Authors:** Jiapeng Liu

2 Mar 2026

PONE-D-26-06689

Low-Cost Thermoelectric Temperature Management for Autonomous Sensor Nodes in Extreme Desert Climates

PLOS One

Dear Dr. Jubair,

Thank you for submitting your manuscript to PLOS ONE. After careful consideration, we feel that it has merit but does not fully meet PLOS ONE’s publication criteria as it currently stands. Therefore, we invite you to submit a revised version of the manuscript that addresses the points raised during the review process.

Please submit your revised manuscript by Apr 16 2026 11:59PM. If you will need more time than this to complete your revisions, please reply to this message or contact the journal office at plosone@plos.org. When you're ready to submit your revision, log on to https://www.editorialmanager.com/pone/ and select the 'Submissions Needing Revision' folder to locate your manuscript file.

Please include the following items when submitting your revised manuscript:

- A letter that responds to each point raised by the academic editor and reviewer(s). You should upload this letter as a separate file labeled 'Response to Reviewers'.
- A marked-up copy of your manuscript that highlights changes made to the original version. You should upload this as a separate file labeled 'Revised Manuscript with Track Changes'.
- An unmarked version of your revised paper without tracked changes. You should upload this as a separate file labeled 'Manuscript'.

If you would like to make changes to your financial disclosure, please include your updated statement in your cover letter. Guidelines for resubmitting your figure files are available below the reviewer comments at the end of this letter.

If applicable, we recommend that you deposit your laboratory protocols in protocols.io to enhance the reproducibility of your results. Protocols.io assigns your protocol its own identifier (DOI) so that it can be cited independently in the future. For instructions see: https://journals.plos.org/plosone/s/submission-guidelines#loc-laboratory-protocols. Additionally, PLOS ONE offers an option for publishing peer-reviewed Lab Protocol articles, which describe protocols hosted on protocols.io. Read more information on sharing protocols at https://plos.org/protocols?utm_medium=editorial-email&utm_source=authorletters&utm_campaign=protocols.

We look forward to receiving your revised manuscript.

Kind regards,

Jiapeng Liu, Ph.D.

Academic Editor

PLOS One

**Journal Requirements:**

When submitting your revision, we need you to address these additional requirements.

1. Please ensure that your manuscript meets PLOS ONE's style requirements, including those for file naming. The PLOS ONE style templates can be found at

https://journals.plos.org/plosone/s/file?id=wjVg/PLOSOne_formatting_sample_main_body.pdf and

https://journals.plos.org/plosone/s/file?id=ba62/PLOSOne_formatting_sample_title_authors_affiliations.pdf

2. Please note that PLOS One has specific guidelines on code sharing for submissions in which author-generated code underpins the findings in the manuscript. In these cases, we expect all author-generated code to be made available without restrictions upon publication of the work. Please review our guidelines at https://journals.plos.org/plosone/s/materials-and-software-sharing#loc-sharing-code and ensure that your code is shared in a way that follows best practice and facilitates reproducibility and reuse.

3. Please update your submission to use the PLOS LaTeX template. The template and more information on our requirements for LaTeX submissions can be found at http://journals.plos.org/plosone/s/latex.

4. We noted in your submission details that a portion of your manuscript may have been presented or published elsewhere. “Preprint: https://doi.org/10.22541/au.176782560.00599011/v1.”

Please clarify whether this [conference proceeding or publication] was peer-reviewed and formally published. If this work was previously peer-reviewed and published, in the cover letter please provide the reason that this work does not constitute dual publication and should be included in the current manuscript.

5. We note that your Data Availability Statement is currently as follows:

“All relevant data are within the manuscript and its Supporting Information files.”

Please confirm at this time whether or not your submission contains all raw data required to replicate the results of your study. Authors must share the “minimal data set” for their submission. PLOS defines the minimal data set to consist of the data required to replicate all study findings reported in the article, as well as related metadata and methods (https://journals.plos.org/plosone/s/data-availability#loc-minimal-data-set-definition).

For example, authors should submit the following data:

- The values behind the means, standard deviations and other measures reported;

- The values used to build graphs;

- The points extracted from images for analysis.

Authors do not need to submit their entire data set if only a portion of the data was used in the reported study.

If your submission does not contain these data, please either upload them as Supporting Information files or deposit them to a stable, public repository and provide us with the relevant URLs, DOIs, or accession numbers. For a list of recommended repositories, please see https://journals.plos.org/plosone/s/recommended-repositories.

If there are ethical or legal restrictions on sharing a de-identified data set, please explain them in detail (e.g., data contain potentially sensitive information, data are owned by a third-party organization, etc.) and who has imposed them (e.g., an ethics committee). Please also provide contact information for a data access committee, ethics committee, or other institutional body to which data requests may be sent. If data are owned by a third party, please indicate how others may request data access.

6. Please ensure that you refer to Figures 1, 2, 3, 4, 5, and 7 in your text as, if accepted, production will need this reference to link the reader to the figure.

7. We note that Figure 3 in your submission contain copyrighted images. All PLOS content is published under the Creative Commons Attribution License (CC BY 4.0), which means that the manuscript, images, and Supporting Information files will be freely available online, and any third party is permitted to access, download, copy, distribute, and use these materials in any way, even commercially, with proper attribution. For more information, see our copyright guidelines: http://journals.plos.org/plosone/s/licenses-and-copyright.

We require you to either (1) present written permission from the copyright holder to publish these figures specifically under the CC BY 4.0 license, or (2) remove the figures from your submission:

1. You may seek permission from the original copyright holder of Figure 3 to publish the content specifically under the CC BY 4.0 license.

We recommend that you contact the original copyright holder with the Content Permission Form (http://journals.plos.org/plosone/s/file?id=7c09/content-permission-form.pdf) and the following text:

“I request permission for the open-access journal PLOS ONE to publish XXX under the Creative Commons Attribution License (CCAL) CC BY 4.0 (http://creativecommons.org/licenses/by/4.0/). Please be aware that this license allows unrestricted use and distribution, even commercially, by third parties. Please reply and provide explicit written permission to publish XXX under a CC BY license and complete the attached form.”

Please upload the completed Content Permission Form or other proof of granted permissions as an "Other" file with your submission.

In the figure caption of the copyrighted figure, please include the following text: “Reprinted from [ref] under a CC BY license, with permission from [name of publisher], original copyright [original copyright year].”

2. If you are unable to obtain permission from the original copyright holder to publish these figures under the CC BY 4.0 license or if the copyright holder’s requirements are incompatible with the CC BY 4.0 license, please either i) remove the figure or ii) supply a replacement figure that complies with the CC BY 4.0 license. Please check copyright information on all replacement figures and update the figure caption with source information. If applicable, please specify in the figure caption text when a figure is similar but not identical to the original image and is therefore for illustrative purposes only.

8. We note you have included a table to which you do not refer in the text of your manuscript. Please ensure that you refer to Tables  1.1, 1.2, 2, and 3 in your text; if accepted, production will need this reference to link the reader to the Table.

9. If the reviewer comments include a recommendation to cite specific previously published works, please review and evaluate these publications to determine whether they are relevant and should be cited. There is no requirement to cite these works unless the editor has indicated otherwise.

**Additional Editor Comments:**

The readability of this manuscript is very poor. It lacks detailed descriptions of the system design and in-depth analyses of the validation results. Please supplement performance metrics other than COP.

[Note: HTML markup is below. Please do not edit.]

Reviewers' comments:

Reviewer's Responses to Questions

**Comments to the Author**

1. Is the manuscript technically sound, and do the data support the conclusions?

The manuscript must describe a technically sound piece of scientific research with data that supports the conclusions. Experiments must have been conducted rigorously, with appropriate controls, replication, and sample sizes. The conclusions must be drawn appropriately based on the data presented.

Reviewer #1: No

Reviewer #2: Yes

Reviewer #3: Partly

**********

2. Has the statistical analysis been performed appropriately and rigorously?

Reviewer #1: No

Reviewer #2: N/A

Reviewer #3: Yes

**********

3. Have the authors made all data underlying the findings in their manuscript fully available?

The PLOS Data policy requires authors to make all data underlying the findings described in their manuscript fully available without restriction, with rare exception (please refer to the Data Availability Statement in the manuscript PDF file). The data should be provided as part of the manuscript or its supporting information, or deposited to a public repository. For example, in addition to summary statistics, the data points behind means, medians and variance measures should be available. If there are restrictions on publicly sharing data—e.g. participant privacy or use of data from a third party—those must be specified.

Reviewer #1: Yes

Reviewer #2: No

Reviewer #3: Yes

**********

4. Is the manuscript presented in an intelligible fashion and written in standard English?

PLOS ONE does not copyedit accepted manuscripts, so the language in submitted articles must be clear, correct, and unambiguous. Any typographical or grammatical errors should be corrected at revision, so please note any specific errors here.

Reviewer #1: Yes

Reviewer #2: No

Reviewer #3: Yes

**********

5. Review Comments to the Author

Please use the space provided to explain your answers to the questions above. You may also include additional comments for the author, including concerns about dual publication, research ethics, or publication ethics. (Please upload your review as an attachment if it exceeds 20,000 characters)

Reviewer #1: In the manuscript "Low-Cost Thermoelectric Temperature Management for Autonomous Sensor Nodes in Extreme Desert Climates",

- introduces an Optimized Passive Thermo-Electric Temperature Regulation System to protect sensitive electronics and batteries.

- uses a solid-state Peltier module for active bi-directional temperature control (both cooling and heating).

-incorporates highly efficient fanless passive heat transfer components to enhance thermal performance.

- reduces power intake and average power consumption by up to 10%.

However, this manuscript lacks

-overall presentation and scientific writing

-The manuscript need through revision

-The figure caption need more description

-The table title need revision

-Some figures need to replot with better quality eg. Fig 7.

Reviewer #2: Review Report

Manuscript Title (requires revision): Low-Cost Thermoelectric Temperature Management for Autonomous Sensor Nodes in Extreme Desert Climates

The manuscript presents an interesting and practically relevant thermal management solution for autonomous sensor nodes operating in extreme desert environments. The integration of a Peltier module with passive, fanless heat dissipation structures is promising, and the emphasis on low cost, locally sourced materials is a notable strength. However, several areas require substantial revision to improve scientific rigor, contextual grounding, and clarity. The manuscript would benefit from deeper engagement with recent literature, clearer articulation of the engineering significance of the results, and a more accurate and representative title.

• The manuscript cites some foundational work but lacks engagement with recent thermoelectric, desert environment, and IoT thermal management literature (2020–2024). This limits the contextual strength of the contribution.

• Please compare your COP and power consumption improvements with recent studies on: Passive/active hybrid cooling for IoT nodes in arid climates, Low power thermoelectric cooling systems, Dust resilient thermal enclosures, and Solar powered thermal management systems

• Several recent works demonstrate improved heat sink geometries, PCM integrated cooling, and radiative cooling coatings. How does OPTERS compare in terms of: Thermal response time, Energy efficiency, and Long term reliability under dust exposure

• Your experimental results show a 10% reduction in average power consumption. How does this compare with other PWM controlled Peltier systems reported in the literature?

• The introduction relies heavily on older or general references. To strengthen the scientific framing, the authors should incorporate recent studies (2020–2024) on: Thermoelectric cooling in IoT and WSN systems, Desert environment electronics reliability, Passive radiative cooling and fanless heat sink designs, Solar powered embedded systems, and Dust proof enclosure engineering

• Add recent work on ultra low power thermal management for remote sensing nodes.

• Include studies on desert specific thermal challenges (e.g., sand ingress, radiative heating, thermal cycling fatigue).

• Clarify the gap in the literature: active thermoelectric cooling for sensor nodes in desert climates remains underexplored—this is a strong point but needs stronger citation support.

• What is the physical mechanism behind the observed 10% reduction in power consumption?

o Is it due to PWM efficiency, improved heat sink geometry, or reduced thermal cycling?

• How does the enclosure design influence thermal resistance and heat flow pathways?

• What is the expected lifetime improvement for electronics and batteries when internal temperature is stabilized at ~20 °C?

• How does dust accumulation affect thermal performance over time?

o Please quantify or discuss expected degradation rates.

• What is the thermal response time (cooling/heating rate) of the system under realistic desert temperature swings?

• Provide a clear thermodynamic explanation of how the passive heat sink geometry enhances Peltier performance.

• Include a more detailed discussion of the COP calculation and its significance.

• Explain the engineering trade offs: weight, cost, thermal inertia, and energy budget.

• Avoid general statements such as “superior solution” unless supported by comparative data. Include limitations: Prototype scale testing, Lack of long term outdoor validation, and Limited dust exposure quantification

• Clarify what is demonstrated versus what is anticipated.

• Strengthen the conclusion by summarizing: Key quantitative findings, Practical implications for desert deployed sensor nodes, Future work (e.g., field trials, optimization of heat sink geometry, integration with PCM or radiative coatings)

The current title does not fully reflect the manuscript’s scope, especially the emphasis on passive heat sink optimization, PWM control, and desert specific challenges.

Suggested Alternatives

• “Optimized Thermoelectric Thermal Regulation for Autonomous Sensor Nodes in Extreme Desert Environments”

• “Fanless Thermoelectric Temperature Control System for Desert Deployed Autonomous Sensor Nodes”

• “Solar Powered Thermoelectric Thermal Management for Dust Resilient Sensor Nodes in Arid Climates”

6. Additional Technical and Editorial Comments

• Figures require clearer labeling, higher resolution, and consistent formatting.

• The circuit diagram should be redrawn professionally.

• Some sections contain duplicated or repeated sentences (e.g., temperature descriptions).

• Ensure consistent use of units (°C, W, g/cm³).

• Improve grammar and remove typographical errors throughout the manuscript.

• The methodology section should include a block diagram summarizing system architecture.

• Provide a cost breakdown to support the “low cost” claim.

Reviewer #3: General Appreciation:

The manuscript presents a technically sound and innovative low-cost thermal management system for autonomous desert sensor nodes. The integration of Peltier modules with PWM control is well-executed and offers a practical solution for remote deployments.

Required Revisions:

1-Ambient Temperature Data: The primary concern is the absence of Ambient Temperature (Environment Temperature) in Tables 1.1 and 1.2. To validate the system's performance in "Extreme Desert Climates," it is essential to record and display the external temperature for each data point to demonstrate the actual thermal gradient achieved against the desert environment.

2-Circuit Schematic: Figure 1 (Circuit Diagram) currently omits the battery and BMS connections. For a system claiming "Autonomous 24/7 operation," a complete schematic including the energy storage interface is necessary to serve as a robust technical reference.

**********

6. PLOS authors have the option to publish the peer review history of their article (what does this mean?). If published, this will include your full peer review and any attached files.

If you choose “no”, your identity will remain anonymous but your review may still be made public.

**Do you want your identity to be public for this peer review?**   For information about this choice, including consent withdrawal, please see our Privacy Policy.

Reviewer #1: No

Reviewer #2: No

Reviewer #3: No

**********

[NOTE: If reviewer comments were submitted as an attachment file, they will be attached to this email and accessible via the submission site. Please log into your account, locate the manuscript record, and check for the action link "View Attachments". If this link does not appear, there are no attachment files.]

To ensure your figures meet our technical requirements, please review our figure guidelines: https://journals.plos.org/plosone/s/figures

You may also use PLOS’s free figure tool, NAAS, to help you prepare publication quality figures: https://journals.plos.org/plosone/s/figures#loc-tools-for-figure-preparation.

NAAS will assess whether your figures meet our technical requirements by comparing each figure against our figure specifications.

# Author comment: Author response to Decision Letter 1

**DOI:** 10.1371/journal.pone.0352185.r002

6 Apr 2026

We thank the Academic Editor and the Reviewers for their time, constructive feedback, and valuable insights. We have carefully revised the manuscript to address all concerns. Below is our point-by-point response.

Response to Journal Requirements

1 & 3. PLOS ONE Style and LaTeX Template: We have updated the entire manuscript to use the official PLOS ONE LaTeX template and ensured all formatting complies with the journal's style guidelines.

2. Code Sharing: Our system utilizes standard PWM control logic on an ESP32 microcontroller. All algorithmic flow is documented in Figure 3.

4. Preprint/Dual Publication: This has been addressed in our Cover Letter. The preprint is an early, non-peer-reviewed version of this work.

5. Data Availability: We confirm that all raw data required to replicate the results are fully included within the manuscript text and tables (specifically Tables 2, 3, 4, 5, and 6).

6 & 8. Figure and Table Citations: Due to the addition of new diagrams and tables, the numbering has changed. We have meticulously verified that all current figures (Figures 1 through 8) and tables (Tables 1 through 6) are explicitly cited in the text.

7. Figure 3 Copyright: We have completely removed the copyrighted image and replaced it with an original, author-generated diagram that complies with the CC BY 4.0 license.

Response to Academic Editor

Comment: The readability of this manuscript is very poor. It lacks detailed descriptions of the system design and in-depth analyses of the validation results. Please supplement performance metrics other than COP.

Response: We have thoroughly rewritten the manuscript to elevate the scientific tone and readability. To provide detailed descriptions of the system design, we added a new System Architecture Block Diagram (Figure 1) and a fully detailed Circuit Diagram (Figure 2). To supplement performance metrics beyond COP, we added Table 6, which now includes maximum temperature overshoot (1.8°C), undershoot (1.2°C), settling time (4.6 min), steady-state temperature standard deviation (±0.42°C), and dust ingress impact.

Response to Reviewer 1

Comment: Lacks overall presentation and scientific writing. The manuscript needs thorough revision.

Response: The entire manuscript has been heavily proofread and revised to improve grammar, flow, and scientific rigor.

Comment: The figure captions need more description. The table titles need revision. Response: We have expanded all figure captions and table titles to be fully descriptive and self-contained so readers can understand them without searching the main text.

Comment: Some figures need to replot with better quality eg. Fig 7.

Response: We replotted the COP curve (previously Figure 7) to ensure high resolution, clear labeling, and publication-ready quality.

Response to Reviewer 2

Comment: The current title does not fully reflect the manuscript’s scope... Suggested Alternatives...

Response: We agree. We updated the title to "Optimized Passive Thermoelectric Temperature Regulation for Dust-Resilient Sensor Nodes in Extreme Desert Climates" to better reflect the engineering scope and environment.

Comment: The manuscript cites some foundational work but lacks engagement with recent thermoelectric, desert environment, and IoT thermal management literature (2020–2024).

Response: We have heavily revised Section 2 (Literature Review) to incorporate recent literature from 2020 to 2024. We added discussions on hybrid cooling, PCM integration, radiative cooling coatings, and low-power thermoelectric systems.

Comment: What is the physical mechanism behind the observed 10% reduction in power consumption?

Response: We expanded Section 6 (Discussion) to clarify this. The reduction is driven by a combination of PWM efficiency minimizing parasitic Joule heating, the zero-power nature of our fanless passive heat sinks, and tight thermal regulation preventing high-power corrective spikes.

Comment: How does dust accumulation affect thermal performance over time? What is the expected lifetime improvement? What is the thermal response time?

Response: We created a brand new section, "Section 5: Environmental Resilience, Dynamic Response, and Lifetime Implications", to address these exact questions. We also quantified the rapid thermal response time (4.6 minutes to reach 20°C) and the negligible impact of dust after our 72-hour simulation in Table 6.

Comment: Avoid general statements such as “superior solution” unless supported by comparative data. Include limitations... Strengthen the conclusion…

Response: We removed subjective phrases like "superior solution". We also rewrote the Conclusion to clearly state our quantitative findings, practical implications, and explicit limitations (e.g., prototype scale, need for long-term outdoor validation).

Comment: Provide a cost breakdown to support the “low cost” claim. The methodology section should include a block diagram summarizing system architecture. Ensure consistent use of units.

Response: We added a detailed cost breakdown in Table 1, showing the $65 total cost. We added the requested System Architecture Block Diagram as Figure 1. We also standardized all units (°C, W) throughout the text.

Response to Reviewer 3

Comment: Ambient Temperature Data: The primary concern is the absence of Ambient Temperature (Environment Temperature) in Tables 1.1 and 1.2.

Response: We have added a dedicated "Ambient Temperature" column to our cooling data tables (now labeled Table 2 and Table 3) to properly demonstrate the thermal gradient.

Comment: Circuit Schematic: Figure 1 (Circuit Diagram) currently omits the battery and BMS connections. For a system claiming "Autonomous 24/7 operation," a complete schematic including the energy storage interface is necessary.

Response: Thank you for pointing this out. We completely redrew the schematic (now Figure 2) to explicitly include the Li-ion battery pack and the MPPT solar charge controller, which handles the BMS functionality.

Attachment Submitted filename: Reviewer Comments PLOS One.pdf

# Peer review: Decision Letter 1

**DOI:** 10.1371/journal.pone.0352185.r003

**Authors:** Jiapeng Liu

21 Apr 2026

PONE-D-26-06689R1

Optimized Passive Thermoelectric Temperature Regulation for Dust-Resilient Sensor Nodes in Extreme Desert Climates

PLOS One

Dear Dr. Jubair,

Thank you for submitting your manuscript to PLOS ONE. After careful consideration, we feel that it has merit but does not fully meet PLOS ONE’s publication criteria as it currently stands. Therefore, we invite you to submit a revised version of the manuscript that addresses the points raised during the review process.

==============================

**ACADEMIC EDITOR:**   More explanations are required regarding the rationality of the experimental validation design.

==============================

Please submit your revised manuscript by Jun 05 2026 11:59PM. If you will need more time than this to complete your revisions, please reply to this message or contact the journal office at plosone@plos.org. When you're ready to submit your revision, log on to https://www.editorialmanager.com/pone/ and select the 'Submissions Needing Revision' folder to locate your manuscript file.

Please include the following items when submitting your revised manuscript:

- A letter that responds to each point raised by the academic editor and reviewer(s). You should upload this letter as a separate file labeled 'Response to Reviewers'.
- A marked-up copy of your manuscript that highlights changes made to the original version. You should upload this as a separate file labeled 'Revised Manuscript with Track Changes'.
- An unmarked version of your revised paper without tracked changes. You should upload this as a separate file labeled 'Manuscript'.

If you would like to make changes to your financial disclosure, please include your updated statement in your cover letter. Guidelines for resubmitting your figure files are available below the reviewer comments at the end of this letter.

If applicable, we recommend that you deposit your laboratory protocols in protocols.io to enhance the reproducibility of your results. Protocols.io assigns your protocol its own identifier (DOI) so that it can be cited independently in the future. For instructions see: https://journals.plos.org/plosone/s/submission-guidelines#loc-laboratory-protocols. Additionally, PLOS ONE offers an option for publishing peer-reviewed Lab Protocol articles, which describe protocols hosted on protocols.io. Read more information on sharing protocols at https://plos.org/protocols?utm_medium=editorial-email&utm_source=authorletters&utm_campaign=protocols.

As the corresponding author, your ORCID iD is verified in the submission system and will appear in the published article. PLOS supports the use of ORCID, and we encourage all coauthors to register for an ORCID iD and use it as well. Please encourage your coauthors to verify their ORCID iD within the submission system before final acceptance, as unverified ORCID iDs will not appear in the published article. *Only*   the individual author can complete the verification step; PLOS staff *cannot*   verify ORCID iDs on behalf of authors.

We look forward to receiving your revised manuscript.

Kind regards,

Jiapeng Liu, Ph.D.

Academic Editor

PLOS One

Journal Requirements:

If the reviewer comments include a recommendation to cite specific previously published works, please review and evaluate these publications to determine whether they are relevant and should be cited. There is no requirement to cite these works unless the editor has indicated otherwise.

[Note: HTML markup is below. Please do not edit.]

Reviewers' comments:

Reviewer's Responses to Questions

**Comments to the Author**

1. If the authors have adequately addressed your comments raised in a previous round of review and you feel that this manuscript is now acceptable for publication, you may indicate that here to bypass the “Comments to the Author” section, enter your conflict of interest statement in the “Confidential to Editor” section, and submit your "Accept" recommendation.

Reviewer #1: All comments have been addressed

Reviewer #2: All comments have been addressed

Reviewer #3: (No Response)

**********

2. Is the manuscript technically sound, and do the data support the conclusions?

The manuscript must describe a technically sound piece of scientific research with data that supports the conclusions. Experiments must have been conducted rigorously, with appropriate controls, replication, and sample sizes. The conclusions must be drawn appropriately based on the data presented.

Reviewer #1: Yes

Reviewer #2: Yes

Reviewer #3: Partly

**********

3. Has the statistical analysis been performed appropriately and rigorously?

Reviewer #1: Yes

Reviewer #2: I Don't Know

Reviewer #3: No

**********

4. Have the authors made all data underlying the findings in their manuscript fully available?

The PLOS Data policy requires authors to make all data underlying the findings described in their manuscript fully available without restriction, with rare exception (please refer to the Data Availability Statement in the manuscript PDF file). The data should be provided as part of the manuscript or its supporting information, or deposited to a public repository. For example, in addition to summary statistics, the data points behind means, medians and variance measures should be available. If there are restrictions on publicly sharing data—e.g. participant privacy or use of data from a third party—those must be specified.

Reviewer #1: (No Response)

Reviewer #2: Yes

Reviewer #3: No

**********

5. Is the manuscript presented in an intelligible fashion and written in standard English?

PLOS ONE does not copyedit accepted manuscripts, so the language in submitted articles must be clear, correct, and unambiguous. Any typographical or grammatical errors should be corrected at revision, so please note any specific errors here.

Reviewer #1: (No Response)

Reviewer #2: Yes

Reviewer #3: No

**********

6. Review Comments to the Author

Please use the space provided to explain your answers to the questions above. You may also include additional comments for the author, including concerns about dual publication, research ethics, or publication ethics. (Please upload your review as an attachment if it exceeds 20,000 characters)

Reviewer #1: The authors have revised the manuscript based on the previous comments. The revised version is acceptable.

Reviewer #2: The revised manuscript has been carefully evaluated, and it is evident that the authors have effectively addressed the required revisions and responded thoroughly to the majority of the reviewers’ comments. These efforts have led to a notable improvement in the clarity, rigor, and overall quality of the work. In view of these substantial enhancements, I believe that the manuscript now satisfies the standards of the journal and is suitable for publication.

Reviewer #3: The manuscript presents an interesting approach to thermal management in arid environments; however, specific technical updates are necessary to ensure the research is fully supported by the presented data and diagrams.

1. Completeness of the Technical Schematic (Figure 1):

• The system is described as capable of "Autonomous 24/7 operation" through solar harvesting and battery storage.

• However, the circuit diagram in Figure 1 currently omits the battery pack and its interface.

• To serve as a robust technical reference, the schematic must be updated to include the actual wiring for the battery, MPPT charge controller, and Battery Management System (BMS) mentioned in the methodology.

2. Inclusion of Ambient Temperature Data in Tables 1.1 & 1.2:

• The primary objective of the study is to validate performance in "Extreme Desert Climates".

• Currently, Tables 1.1 and 1.2 list temperatures for the hot and cold sides of the module but do not include the Ambient (Environmental) Temperature for each recorded data point in cooling mode.

• Providing the external temperature baseline for every experiment is essential to demonstrate the actual thermal gradient achieved and to verify the reported Coefficient of Performance (COP).

These technical clarifications are necessary to align the experimental results with the manuscript's stated objectives and to ensure the reproducibility of the system.

**********

7. PLOS authors have the option to publish the peer review history of their article (what does this mean?). If published, this will include your full peer review and any attached files.

If you choose “no”, your identity will remain anonymous but your review may still be made public.

**Do you want your identity to be public for this peer review?**   For information about this choice, including consent withdrawal, please see our Privacy Policy.

Reviewer #1: No

Reviewer #2: No

Reviewer #3: No

**********

[NOTE: If reviewer comments were submitted as an attachment file, they will be attached to this email and accessible via the submission site. Please log into your account, locate the manuscript record, and check for the action link "View Attachments". If this link does not appear, there are no attachment files.]

To ensure your figures meet our technical requirements, please review our figure guidelines: https://journals.plos.org/plosone/s/figures

You may also use PLOS’s free figure tool, NAAS, to help you prepare publication quality figures: https://journals.plos.org/plosone/s/figures#loc-tools-for-figure-preparation.

NAAS will assess whether your figures meet our technical requirements by comparing each figure against our figure specifications.

# Author comment: Author response to Decision Letter 2

**DOI:** 10.1371/journal.pone.0352185.r004

29 May 2026

Response to Academic Editor

Comment: More explanations are required regarding the rationality of the experimental validation design.

Our Response: We thank the Academic Editor for highlighting the need for further clarification on the rationality of our experimental validation design. We have incorporated a new paragraph into Section 3.3, "Experimental Setup and Real-World Simulation," to explicitly address this point. The added text provides a detailed explanation of why our laboratory-based simulation approach is logical and how it accurately mimics real desert conditions.

The new paragraph emphasizes:

Controlled Environment: The rationale for using controlled laboratory settings to isolate and precisely manage key environmental stressors, ensuring repeatable and quantifiable results.

Simulation of Diurnal Temperature Swings: How low-resistance heating coils were used to generate internal temperature spikes (exceeding 100°C, simulating internal heat loads from solar radiation or component self-heating) and how ambient temperatures were varied (from 3°C to 45-60°C) to comprehensively assess bidirectional thermal regulation.

Dust Ingress Simulation: The use of a commercially available fine particulate matter simulant (with densities up to 2.3 g/cm³) to directly evaluate the dust-resistant enclosure and its impact on heat dissipation and sensor reliability.

Logical Representation: A concluding statement affirming that, despite the inherent limitations of any laboratory simulation, our systematic control of these parameters provides a logical and accurate representation of the primary thermal and particulate challenges in arid climates, thus robustly validating the OPTERS system's performance.

We believe this expanded explanation thoroughly addresses the editor's comment and provides a stronger foundation for the experimental results presented in the manuscript.

Response to Reviewer #3

We thank Reviewer #3 for their insightful comments and constructive feedback, which have significantly helped us improve the clarity and technical robustness of our manuscript. We are pleased to hear that the revised version is suitable for publication according to the other two reviewers. We have carefully addressed the points raised.

Reviewer Comment 1: Completeness of the Technical Schematic (Figure 1)

Original Comment: "The system is described as capable of 'Autonomous 24/7 operation' through solar harvesting and battery storage. However, the circuit diagram in Figure 1 currently omits the battery pack and its interface. To serve as a robust technical reference, the schematic must be updated to include the actual wiring for the battery, MPPT charge controller, and Battery Management System (BMS) mentioned in the methodology."

Our Response: We appreciate the reviewer's keen observation regarding the completeness of Figure 1. We agree that a comprehensive technical schematic is crucial for a robust technical reference, especially given the system's autonomous 24/7 operation capabilities. We confirm that the updated Figure 1 (now Figure 2 in the revised manuscript, as Figure 1 is now the system architecture block diagram) has been revised to include the detailed wiring for the solar panel, MPPT solar charge controller, Li-ion battery pack, and the Battery Management System (BMS). This updated schematic now clearly illustrates the electrical connections and control pathways for power harvesting, storage, and distribution, providing a more complete and accurate representation of the OPTERS system's power management architecture. We believe this enhancement significantly improves the technical clarity and utility of the figure for readers.

Reviewer Comment 2: Inclusion of Ambient Temperature Data in Tables 1.1 & 1.2

Original Comment: "Currently, Tables 1.1 and 1.2 list temperatures for the hot and cold sides of the module but do not include the Ambient (Environmental) Temperature for each recorded data point in cooling mode. Providing the external temperature baseline for every experiment is essential to demonstrate the actual thermal gradient achieved and to verify the reported Coefficient of Performance (COP)."

Our Response: We sincerely thank the reviewer for highlighting this critical point. We completely agree that the external temperature baseline is essential to demonstrate the actual thermal gradient and validate the COP under simulated desert conditions. Please note that during the revision process, the original Tables 1.1 and 1.2 were renamed and reformatted as Table 2 and Table 3 in the current manuscript. As requested, we have ensured that the Ambient (Environmental) Temperature corresponding to every recorded data point is explicitly included as a dedicated column in both Table 2 (real-time data) and Table 3 (averaged data). We hope this fully clarifies the thermal gradients achieved during our experimental validation and satisfies the requirement for reproducibility.

Attachment Submitted filename: __Response to the reviewers.docx

# Peer review: Decision Letter 2

**DOI:** 10.1371/journal.pone.0352185.r005

**Authors:** Jiapeng Liu

8 Jun 2026

Optimized Passive Thermoelectric Temperature Regulation for Dust-Resilient Sensor Nodes in Extreme Desert Climates

PONE-D-26-06689R2

Dear Dr. Jubair,

We’re pleased to inform you that your manuscript has been judged scientifically suitable for publication and will be formally accepted for publication once it meets all outstanding technical requirements.

Within one week, you’ll receive an e-mail detailing the required amendments. When these have been addressed, you’ll receive a formal acceptance letter and your manuscript will be scheduled for publication.

An invoice will be generated when your article is formally accepted. Please note, if your institution has a publishing partnership with PLOS and your article meets the relevant criteria, all or part of your publication costs will be covered. Please make sure your user information is up-to-date by logging into Editorial Manager at Editorial Manager® and clicking the ‘Update My Information' link at the top of the page. For questions related to billing, please contact billing support.

If your institution or institutions have a press office, please notify them about your upcoming paper to help maximize its impact. If they’ll be preparing press materials, please inform our press team as soon as possible -- no later than 48 hours after receiving the formal acceptance. Your manuscript will remain under strict press embargo until 2 pm Eastern Time on the date of publication. For more information, please contact onepress@plos.org.

Kind regards,

Jiapeng Liu, Ph.D.

Academic Editor

PLOS One

Additional Editor Comments (optional):

Reviewers' comments:

Reviewer's Responses to Questions

**Comments to the Author**

1. If the authors have adequately addressed your comments raised in a previous round of review and you feel that this manuscript is now acceptable for publication, you may indicate that here to bypass the “Comments to the Author” section, enter your conflict of interest statement in the “Confidential to Editor” section, and submit your "Accept" recommendation.

Reviewer #3: All comments have been addressed

**********

2. Is the manuscript technically sound, and do the data support the conclusions?

The manuscript must describe a technically sound piece of scientific research with data that supports the conclusions. Experiments must have been conducted rigorously, with appropriate controls, replication, and sample sizes. The conclusions must be drawn appropriately based on the data presented.

Reviewer #3: Yes

**********

3. Has the statistical analysis been performed appropriately and rigorously?

Reviewer #3: Yes

**********

4. Have the authors made all data underlying the findings in their manuscript fully available?

The PLOS Data policy requires authors to make all data underlying the findings described in their manuscript fully available without restriction, with rare exception (please refer to the Data Availability Statement in the manuscript PDF file). The data should be provided as part of the manuscript or its supporting information, or deposited to a public repository. For example, in addition to summary statistics, the data points behind means, medians and variance measures should be available. If there are restrictions on publicly sharing data—e.g. participant privacy or use of data from a third party—those must be specified.

Reviewer #3: Yes

**********

5. Is the manuscript presented in an intelligible fashion and written in standard English?

PLOS ONE does not copyedit accepted manuscripts, so the language in submitted articles must be clear, correct, and unambiguous. Any typographical or grammatical errors should be corrected at revision, so please note any specific errors here.

Reviewer #3: Yes

**********

6. Review Comments to the Author

Please use the space provided to explain your answers to the questions above. You may also include additional comments for the author, including concerns about dual publication, research ethics, or publication ethics. (Please upload your review as an attachment if it exceeds 20,000 characters)

Reviewer #3: I highly commend the authors for their diligent efforts in addressing all previous feedback. The manuscript has been significantly strengthened, making it fully ready for publication. The implementation of the PWM control is well-rationalized, and the empirical data fully validate the system's cooling efficiency and power reduction. Furthermore, the updated Section 3.3 successfully clarifies the rationale behind the laboratory-based simulations, while reporting the mean values and standard deviations ensures data repeatability. The authors are also adding the Ambient Temperature column to verify the thermal gradients. The manuscript is written in clear standard English, and the upgraded technical schematics serve as excellent references. All comments have been comprehensively addressed, and I highly recommend this manuscript for immediate acceptance.

**********

7. PLOS authors have the option to publish the peer review history of their article (what does this mean?). If published, this will include your full peer review and any attached files.

If you choose “no”, your identity will remain anonymous but your review may still be made public.

**Do you want your identity to be public for this peer review?**   For information about this choice, including consent withdrawal, please see our Privacy Policy.

Reviewer #3: No

**********

# Editor report: Acceptance letter

**DOI:** 10.1371/journal.pone.0352185.r006

**Authors:** Jiapeng Liu

PONE-D-26-06689R2

PLOS One

Dear Dr. Jubair,

I'm pleased to inform you that your manuscript has been deemed suitable for publication in PLOS One. Congratulations! Your manuscript is now being handed over to our production team.

At this stage, our production department will prepare your paper for publication. This includes ensuring the following:

* All references, tables, and figures are properly cited

* All relevant supporting information is included in the manuscript submission,

* There are no issues that prevent the paper from being properly typeset

You will receive further instructions from the production team, including instructions on how to review your proof when it is ready. Please keep in mind that we are working through a large volume of accepted articles, so please give us a few days to review your paper and let you know the next and final steps.

Lastly, if your institution or institutions have a press office, please let them know about your upcoming paper now to help maximize its impact. If they'll be preparing press materials, please inform our press team within the next 48 hours. Your manuscript will remain under strict press embargo until 2 pm Eastern Time on the date of publication. For more information, please contact onepress@plos.org.

You will receive an invoice from PLOS for your publication fee after your manuscript has reached the completed accept phase. If you receive an email requesting payment before acceptance or for any other service, this may be a phishing scheme. Learn how to identify phishing emails and protect your accounts at https://explore.plos.org/phishing.

If we can help with anything else, please email us at customercare@plos.org.

Thank you for submitting your work to PLOS ONE and supporting open access.

Kind regards,

PLOS ONE Editorial Office Staff

on behalf of

Dr. Jiapeng Liu

Academic Editor

PLOS One
